# Cellular and molecular evidence that synaptic Schwann cells contribute to aging of mouse neuromuscular junctions

**DOI:** 10.1111/acel.13981

**Published:** 2023-09-28

**Authors:** Robert Louis Hastings, Mary Flordelys Avila, Emma Suneby, Devin Juros, Anson O'Young, Jason Peres da Silva, Gregorio Valdez

**Affiliations:** ^1^ Department of Molecular Biology, Cell Biology and Biochemistry Brown University Providence Rhode Island USA; ^2^ Pathobiology Graduate Program Brown University Providence Rhode Island USA; ^3^ Molecular Biology, Cell Biology, & Biochemistry Graduate Program Brown University Providence Rhode Island USA; ^4^ Center for Translational Neuroscience, Robert J. and Nancy D. Carney Institute for Brain Science, and Center on the Biology of Aging Brown University Providence Rhode Island USA

**Keywords:** aging, NMJ, perisynaptic Schwann cell, synapses, synaptic glia, terminal Schwann cell

## Abstract

Age‐induced degeneration of the neuromuscular junction (NMJ) is associated with motor dysfunction and muscle atrophy. While the impact of aging on the NMJ presynapse and postsynapse is well‐documented, little is known about the changes perisynaptic Schwann cells (PSCs), the synaptic glia of the NMJ, undergo during aging. Here, we examined PSCs in young, middle‐aged, and old mice in three muscles with different susceptibility to aging. Using light and electron microscopy, we found that PSCs acquire age‐associated cellular features either prior to or at the same time as the onset of NMJ degeneration. Notably, we found that aged PSCs fail to completely cap the NMJ even though they are more abundant in old compared with young mice. We also found that aging PSCs form processes that either intrude into the synaptic cleft or guide axonal sprouts to innervate other NMJs. We next profiled the transcriptome of PSCs and other Schwann cells (SCs) to identify mechanisms altered in aged PSCs. This analysis revealed that aged PSCs acquire a transcriptional pattern previously shown to promote phagocytosis that is absent in other SCs. It also showed that aged PSCs upregulate unique pro‐inflammatory molecules compared to other aged SCs. Interestingly, neither synaptogenesis genes nor genes that are typically upregulated by repair SCs were induced in aged PSCs or other SCs. These findings provide insights into cellular and molecular mechanisms that could be targeted in PSCs to stave off the deleterious effects of aging on NMJs.

## Graphical abstract

Age‐induced degeneration of the neuromuscular junction (NMJ) is associated with motor dysfunction and muscle atrophy. We found that the synaptic glia of the NMJ, known as perisynaptic Schwann cells (PSCs), are more abundant in aged mice, fail to completely cap the NMJ, and form processes that either intrude into the synaptic cleft or extend to neighboring NMJs. These cellular changes are accompanied by increased expression of genes involved in phagocytosis and cytokine signaling.

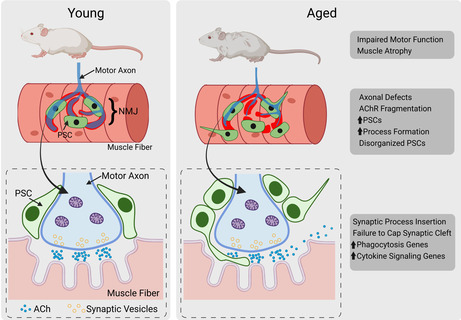

**Abbreviations**
ALS: amyotrophic lateral sclerosis
AS: axon terminal sprouting
Bleb: axonal blebbing
CNS: central nervous system
DEG: differentially expressed gene
EDL: extensor digitorum longus
EM: electron microscopy
EOM: extra‐ocular muscle
F4/80: Adgre1
FACS: fluorescence‐activated cell sorting
fBTX: Alexa Fluor555‐conjugated alpha bungarotoxin
Frag: nAChR fragmentation
IPA: Ingenuity Pathway Analysis
Lep: leptin
MA: middle aged
nAChR: nicotinicacetylcholine receptor
NMJ: neuromuscular junction
NNRI: nearest neighbor regularity index
PFA: paraformaldehyde
PI: polyinnervation
PSC: perisynaptic Schwann cell
RT: room temperature
SC: Schwann cell
SEM: standard error of the mean
SMA: spinal muscular atrophy
STM: sternomastoid
TA: tibialis anterior
YA: young adult

## 1 INTRODUCTION

Aging acutely impacts the motor system, where degeneration of the synapses between motor neurons and skeletal muscles, known as neuromuscular junctions (NMJs), contribute to motor deficits in aged individuals (Cruz‐Jentoft & Sayer, 2019; Deschenes et al., 2010; Rudolf et al., 2014; Soendenbroe et al., 2021; Taetzsch & Valdez, 2018; Vandervoort, 2002). For instance, studies of NMJs in rodents and humans have shown that NMJ degeneration progressively increases with age (Soendenbroe et al., 2021; Taetzsch & Valdez, 2018), reaching levels of over 20% in old mice (Chai et al., 2011). This erosion of healthy NMJs during the early stages of aging is thought to precede motor neuron death, cause muscle atrophy, and negatively impact motor function (Jones et al., 2022; Soendenbroe et al., 2021; Taetzsch & Valdez, 2018). Therefore, therapies that target the NMJ could serve as an early prophylaxis for motor function impairments during aging as well as for motor diseases such as amyotrophic lateral sclerosis (ALS), spinal muscular atrophy (SMA), and the spectrum of myasthenia gravis.

While much has been learned about the cellular, molecular, and functional changes that impact the NMJ pre‐ and post‐synapse (Cruz‐Jentoft & Sayer, 2019; Deschenes et al., 2010; Rudolf et al., 2014; Soendenbroe et al., 2021; Taetzsch & Valdez, 2018; Vandervoort, 2002), little is known about how aging impacts the specialized synaptic glia that reside at the NMJ, known as perisynaptic Schwann cells (PSCs) (Fuertes‐Alvarez & Izeta, 2021). PSCs are specialized, nonmyelinating Schwann cells (SCs) associated exclusively with the NMJ (Alvarez‐Suarez et al., 2020; Darabid et al., 2014; Griffin & Thompson, 2008; Ko & Robitaille, 2015). These cells create a barrier that insulates the presynapse and synaptic cleft of the NMJ from nonsynaptic regions of muscles (Boaro et al., 1998; Darabid et al., 2014; Wokke et al., 1990). Similar to astrocytes and microglia, the synaptic glia of the central nervous system (CNS), PSCs perform a number of supportive roles that are increasingly important to the health of the NMJ as it ages, including modulation of cholinergic transmission, synaptic remodeling, phagocytosis of injured axons, and synaptic pruning (Alvarez‐Suarez et al., 2020; Darabid et al., 2014; Griffin & Thompson, 2008; Ko & Robitaille, 2015; Santosa et al., 2018). They also guide motor axons to reinnervate vacated postsynaptic sites by forming cellular processes that guide sprouting motor axons to nearby denervated NMJs (Love & Thompson, 1999; Son & Thompson, 1995a; Son & Thompson, 1995b). Highlighting their importance at NMJs, ablating PSCs leads to NMJ alterations during development and in adulthood (Hastings et al., 2020; Reddy et al., 2003).

Aberrant changes in PSCs have been linked to NMJ degeneration. In a mouse model of ALS, PSCs remain at NMJs but lose their ability to detect synaptic transmission and to guide axons to denervated NMJs (Arbour et al., 2015, 2017; Martineau et al., 2020) as the disease progresses. There is also evidence that increasing the number of PSCs in young adult mice results in fragmentation of the pretzel‐like structure of the NMJ postsynapse (Hastings et al., 2020; Lee et al., 2016), a morphological hallmark of aged rodent NMJs (Valdez et al., 2010). However, it is far less clear when and how aging affects PSCs, or how such changes in PSCs impact NMJs. A number of light and electron microscopy studies in humans and rodents have observed age‐related morphological changes in PSCs, including incomplete synaptic coverage (Chai et al., 2011; Kawabuchi et al., 2001) and insertion of processes into the synaptic cleft (Boaro et al., 1998; Wokke et al., 1990). While these studies did not quantify PSC numbers, they were clearly present at aged NMJs. More recent studies have reported no change in PSC number with age (Willows et al., 2023) or significant declines in PSC number during aging, starting as early as middle‐age, in mice (Ikemoto‐Uezumi et al., 2022; Snyder‐Warwick et al., 2018). Thus, there are contradictory findings about the effect of aging on the morphology and survival of PSCs.

In this study, we defined the impact of aging on PSCs in mice. Using light and electron microscopy, we found that PSCs are more abundant yet fail to completely cover the presynaptic region of aged NMJs. Aged PSCs also form processes that either intrude into the synaptic cleft or extend to adjacent NMJs. We show that while many of these PSC processes were associated with axonal sprouts, the opposite was not the case. Axonal sprouts were almost never found without a PSC process. These findings suggest that PSCs direct motor axons to other NMJs, a phenomenon called “compensatory reinnervation” previously shown to increase the incidence of multiply innervated NMJs, a hallmark of aged skeletal muscle (Boaro et al., 1998), but also proposed to prevent muscle atrophy by innervating muscle fibers denervated during aging (Luff, 1998; Soendenbroe et al., 2021; Vandervoort, 2002). Additional cellular analysis revealed that PSCs lose their organized spatial distribution and engulf the presynapse in a manner that may lead to phagocytosis. To identify genes and pathways involved in PSC aging and with potential roles in NMJ degeneration, we performed RNA sequencing analysis of PSCs alongside other SCs specifically isolated from skeletal muscles using fluorescence‐activated cell sorting (FACS) from young and old mice. Supporting our cellular observations, genes and pathways involved in phagocytosis, inflammation, and intercellular signaling were found altered specifically in aged PSCs. Together, the data in this study reveal cellular and molecular mechanisms altered in aged PSCs associated with NMJ degeneration.

## 2 MATERIALS AND METHODS

A full description of the experimental details is provided in the Appendix S1.

### 2.1 Animals

Thy1‐CFP, RRID:IMSR_JAX:003710 (Feng et al., 2000), and S100β‐GFP, RRID:IMSR_JAX:005621 (Zuo et al., 2004), (S100β‐GFP;thy1‐CFP) mice were used to visualize the axonal terminals of motor neurons and Schwann cells. NG2‐DsRed, RRID:IMSR_JAX:008241 (Zhu et al., 2008), and S100β‐GFP (S100β‐GFP;NG2‐DsRed) were used to isolate PSCs. The S100β‐GFP;thy1‐CFP mice were a generous gift from the Wes Thompson lab. C57BL/6 mice were used for all other immunohistochemistry. “Young adult” mice used for these experiments were 3–5 months old, “middle‐aged” mice were 17 months old, and “old” mice were 23–29 months old. All experiments were carried out under NIH guidelines and Brown University (Protocol# 1905–0013) Institutional Animal Care and Use Committee guidelines.

### 2.2 Immunohistochemistry

For analysis of PSC morphology, mice were perfused intracardially with 4% paraformaldehyde (PFA) and postfixed in 4% PFA overnight. Soleus, extensor digitorum longus (EDL), extraocular muscle (EOM), and sternomastoid (STM) tissue from S100β‐GFP;thy1‐CFP mice were incubated in Alexa Fluor 555 conjugated α‐bungarotoxin (fBTX, Invitrogen #B35451) and DAPI (ThermoFisher D1306, 300 μM) overnight. For immunohistochemistry of whole muscles and muscle cross sections collected from wild‐type mice, the tissue was incubated overnight in primary antibody and for 2 h the following day in secondary antibody, fBTX, and DAPI.

### 2.3 Light microscopy

Images of S100β‐GFP;thy1‐CFP muscles for morphological analysis were taken using a Zeiss LSM 880 confocal microscope with a 40×, 1.3 numerical aperture oil immersion objective. Images of TA cross sections were obtained with a Zeiss LSM 900 confocal microscope with a 63×, 1.4 numerical aperture oil immersion objective. Zeiss Zen software and ImageJ were used to generate z‐stacks and maximum intensity projections.

### 2.4 Transmission electron microscopy

Mice were perfused transcardially with 2% PFA and 3% glutaraldehyde. Extensor digitorum longus muscles were postfixed in 2% PFA/3% glutaraldehyde, stained in 1% osmium tetroxide (Sigma Aldrich, 75632), 1% ferrocyanide (Sigma Aldrich, P3289) followed by 1% uranyl acetate before being dehydrated in graded ethanol. 100 nm cross sections were imaged under a Philips EM410 Transmission Electron Microscope.

### 2.5 Fluorescence activated cell sorting (FACS)

FACS‐isolation of PSCs and other SCs was performed as described previously (Castro et al., 2020).

### 2.6 RNA‐seq

RNA isolation and RNA‐seq on FACS‐isolated cells were performed by Genewiz on six replicates per cell type.

### 2.7 Statistics

Comparisons between two means were made with a 2‐sided, unpaired Student's *t* test, except analysis of PSC counts in relation to NMJ abnormalities, for which a 2‐sided paired Student's *t* test was used. A 1‐way ANOVA with Bonferroni post hoc analysis was used for comparisons of three or more means. A simple linear regression analysis was used to calculate correlation between PSC number and junctional area. An *n* was defined as one animal for all experiments except linear regression analysis where an *n* was defined as one NMJ. Four young adult animals, three middle‐aged animals, and four old animals were used for morphological analysis. One young adult and one old animal were used for electron microscopy. Six young adults and six old mice were analyzed for RNA‐seq. Replicate numbers are provided in the figure legends. The Microsoft Excel Data Analysis plugin (RRID:SCR_016137) and GraphPad Prism (RRID: SCR_002798) were used for statistical analyses. Data are expressed as the mean ± standard error of the mean (SEM).

## 3 RESULTS

### 3.1 More PSCs are found at aged NMJs

**FIGURE 1:**
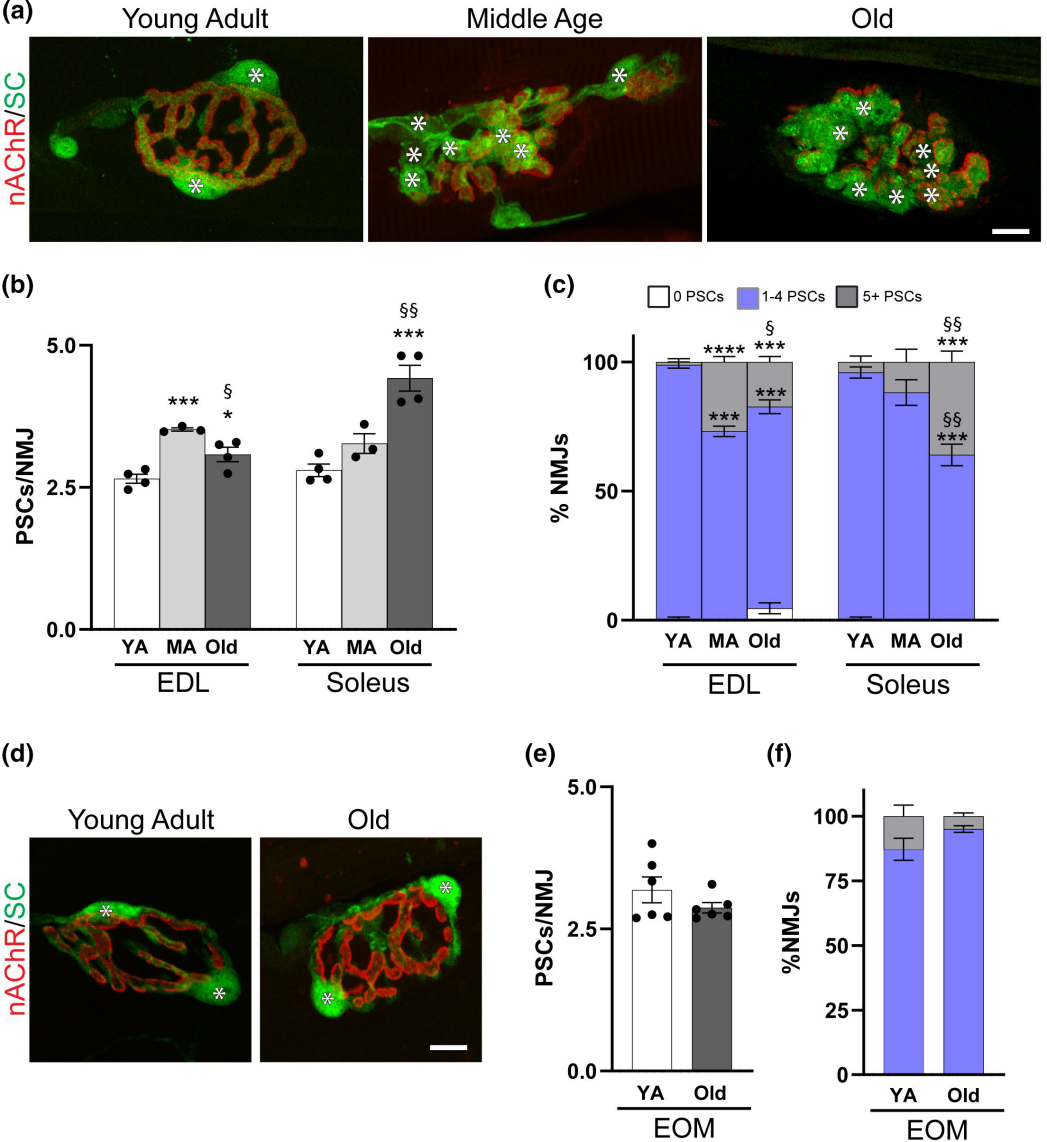
Perisynaptic Schwann cell (PSC) numbers increase in muscles that are susceptible to age‐related NMJ deterioration. (a) Representative images of GFP‐labeled PSCs (green, asterisks) and fBTX‐labeled nAChRs (Red) in the EDL of young adult (3–5 month), middle‐aged (17 month) and old (28–29 month) S100β‐GFP mice. Channel separated images with DAPI counterstain available in Figure S2. (b) Average number of PSCs per NMJ in young adult (YA), middle aged (MA), and old EDL and soleus. (c) Average percentage of NMJs with 0, 1–4, or 5 or more PSCs in young adult (YA), middle aged (MA), and old EDL and soleus. (d) Representative images of GFP‐labeled PSCs (green, asterisks) and fBTX‐labeled nAChRs in the extraocular muscles (EOMs) of young adult (3–5 month) and old (28–29 month) S100β‐GFP mice. (e) Average number of PSCs per NMJ in young adult (YA) and old EOMs. (f) Average percentage of NMJs with 0, 1–4, or 5 or more PSCs in young adult (YA) and old EOMs. *p < 0.05, ***p < 0.001, ****p < 0.0001 versus young adult. ^§^ *p* < 0.05, ^§§^ *p* < 0.01 versus middle aged. One way ANOVA with Bonferroni post hoc (b,c) or unpaired 2‐sided Student's *t* test (e,f). Values represented as mean ± SEM; n = 3–4 (b,c) or 6 (e,f). Scale bars = 10 μm.

We used S100β‐GFP mice to examine the impact of aging on PSC number and morphology. In these transgenic mice, all Schwann cells (SCs) are labeled with GFP but PSCs can be identified based on their unique morphology and location at NMJs in young adult mice (Zuo et al., 2004). First, we confirmed that S100β‐GFP‐positive cells at aged NMJs are indeed PSCs using S100β‐GFP;NG2‐DsRed mice (Figure S1a), in which PSCs can be distinguished from other SCs based on the combined expression of GFP and DsRed (Castro et al., 2020). Additionally, we ascertained that PSCs are present at NMJs of aged wild‐type mice by immunostaining for S100β and NG2 (Figure S1b). We next examined PSCs, in the extensor digitorum longus (EDL) and soleus muscles, two muscles with different muscle fiber types, varying susceptibility to aging, and functional demands (Valdez et al., 2012), in young, middle‐aged and old S100β‐GFP mice. In stark contrast to recently published findings (Ikemoto‐Uezumi et al., 2022; Snyder‐Warwick et al., 2018), our analysis revealed that PSCs were present in the vast majority of NMJs of old mice in the EDL and soleus muscles (Figure 1a and Figure S2). In fact, the average number of PSCs per NMJ was higher in these muscles in old compared with young mice (Figure 1b). In the EDL, the average number of PSCs per NMJ peaked by middle‐age (Figure 1b) owing to the presence of NMJs with five or more PSCs, which were largely absent from the young adult EDL (Figure 1c). While PSCs remained more abundant at NMJs of old compared with young EDL muscles, their average number per NMJ decreased relative to middle‐aged EDL muscles. The decreased number of PSCs between middle‐aged and old EDL resulted from a moderate decrease in the incidence of NMJs with five or more PSCs and a small uptick in NMJs with no detectable PSCs (4.6 ± 2.1%; Figure 1c). In the soleus, the number of PSCs per NMJ became significantly more abundant in old age (Figure 1b). This increase, much as in the EDL, was due to a greater incidence of NMJs containing five or more PSCs in old compared with young adult mice (Figure 1c). These data show that PSCs do not disappear but instead increase during aging in the slow‐twitch soleus and the fast‐twitch EDL muscle. It also revealed that PSCs increase earlier in the EDL compared to the soleus muscle. We also analyzed PSCs in young adult and old extraocular muscles (EOMs), which resist age‐induced degeneration (Figure 1d) (Valdez et al., 2012). Unlike the EDL and soleus muscles, we found no significant change in the average number of PSCs per NMJ in old EOMs (Figure 1e), nor did we find NMJs with abnormally high numbers of PSCs (Figure 1f). These findings indicate that age‐related changes in PSCs may go hand in hand with aging of skeletal muscles and their NMJs.

### 3.2 PSC number correlates with NMJ size in the soleus but not the EDL in aged mice

**FIGURE 2:**
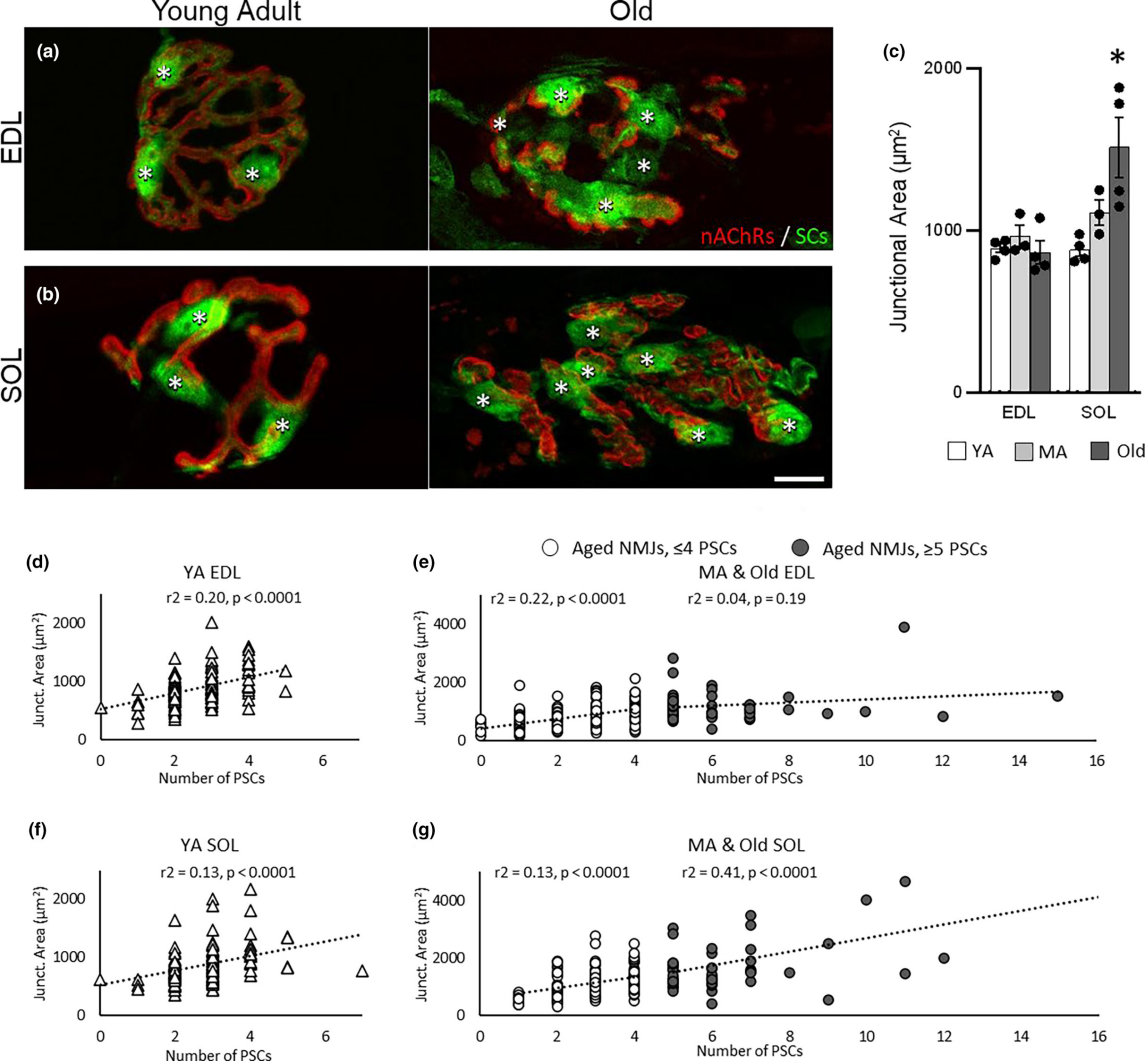
Age‐related increases in PSC numbers correspond with junctional area expansion in the soleus but not EDL. (a,b) Representative images of fBTX‐labeled postsynaptic junctional area and GFP‐labeled PSCs (green, asterisks) in young adult and old EDL (a) and soleus (b) of S100β‐GFP mice. (c) Average junctional area in young adult (YA), middle aged (MA) and old EDL and soleus. Values represented as mean ± SEM and data points represent individual mice; *n* = 3–4. *p < 0.05 versus young adult, one way ANOVA with Bonferroni post hoc. (d,f) Linear regression analysis of junctional area and PSC number in young adult EDL (d) and soleus (f). (e,g) Linear regression analysis of junctional area and PSC number in middle aged and old EDL (e) and soleus (g). Separate analyses performed on NMJs with four or less PSCs and NMJs with five or more PSCs. Data points represent individual NMJs from four mice (young adult) or seven mice (aged). Triangle = Young Adult NMJ. Open circle = Aged NMJ with 4 or less PSCs. Closed (grey) circle = Aged NMJ with 5 or more PSCs. Scale bar = 10 μm.

A well‐understood relationship between PSCs and the NMJ is that the number of PSCs correlates with the junctional area (Love & Thompson, 1998), which is the area calculated by drawing a perimeter around the postsynapse (Figure 2a–c). In the soleus, but not in the EDL, the average junctional area of NMJs increased in old muscle (Figure 2c). In young adult mice, we found the expected strong correlation between the number of PSCs and junctional area in both the EDL and soleus (Figure 2d,f). To examine this relationship during aging, we grouped data from middle‐aged and old mice. We then binned NMJs with four or less PSCs separately from those with five or more PSCs. We found that aged EDL NMJs with four or less PSCs maintained the expected correlation, but this correlation broke down at NMJs with five or more PSCs (Figure 2e). In soleus NMJs, however, we found that PSC number and junctional area correlated regardless of the number of PSCs at a given NMJ (Figure 2g). These data further show that the effects of aging on PSCs and the NMJ differ between the EDL and soleus muscles.

### 3.3 Structured spacing of PSCs breaks down during aging

In healthy, young adult NMJs, PSCs maintain an orderly spatial distribution regardless of number at a given NMJ (Castro et al., 2020). To determine if aging alters the spatial arrangement of PSCs, we used the nearest neighbor regularity index (NNRI) analysis to measure the ordered spacing of PSCs in young and aging NMJs (Cook, 1996). The NNRI evaluates the variability in distances between each PSC and its nearest neighboring PSC by dividing the average nearest neighbor distance by the standard deviation of nearest neighbor distances (Cook, 1996), where increased variability associated with loss of ordered distribution results in lower NNRI values. We found a trend towards lower NNRI values, indicating a loss of ordered spatial distribution, in PSCs of the aged EDL, and a significant decrease in NNRI in PSCs of the aged soleus (Figure S3). These findings suggest that PSCs may lose signals during aging that specify their orderly spatial distribution.

### 3.4 PSCs preferentially increase at NMJs with age‐related features

**FIGURE 3:**
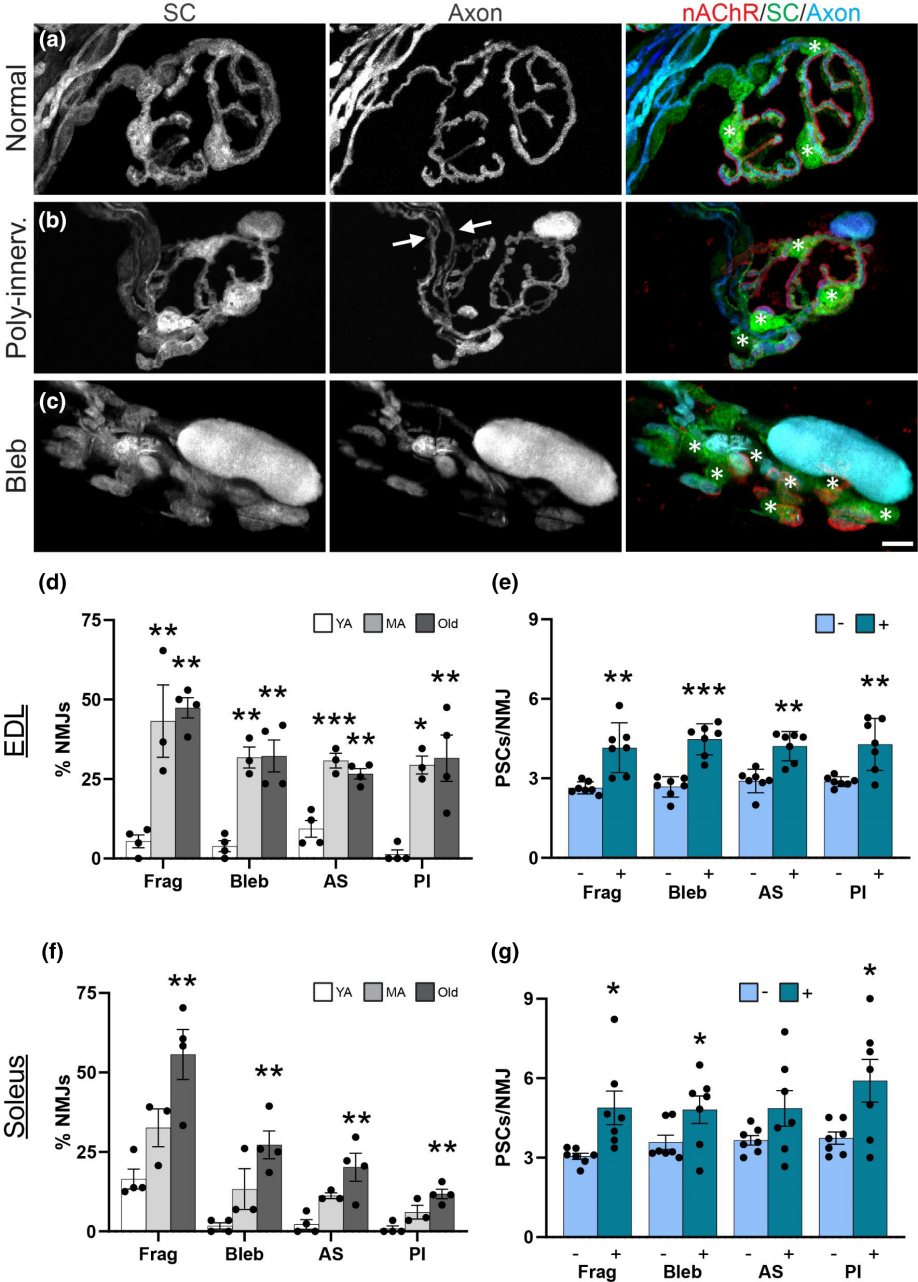
Perisynaptic Schwann cells (PSCs) preferentially increase at NMJs with age‐related features. (a–c) Representative images of GFP‐labeled PSCs (green, asterisks), CFP‐labeled motor axons (blue) and nAChRs (red) at NMJs without axon abnormalities (a), with polyinnervation (b), and with axonal blebs (c) in the EDL of middle‐aged S100β‐GFP;thy1‐CFP mice. Channel separated, wider‐cropped images with DAPI counterstain are available in Figure S4. (d,f) Percentage of NMJs with abnormalities, including nAChR fragmentation (Frag), axonal blebbing (Bleb), axon terminal sprouting (AS), and polyinnervation (PI), in young adult (YA), middle aged (MA), and old EDL (d) and soleus (f). *p < 0.05, **p < 0.01, ***p < 0.001 versus young adult. One way ANOVA with Bonferroni post hoc; n = 3–4. (e,g) Number of PSCs per NMJ with (+) or without (−) abnormalities in middle aged and old EDL (e) and soleus (g). *p < 0.05, **p < 0.01, ***p < 0.001, paired, 2‐sided *t* test. Values represented as mean ± SEM; *n* = 7. Scale bars = 10 μm.

With aging, many NMJs in the soleus and EDL muscles acquire a number of morphological features in both the postsynaptic and presynaptic regions (Valdez et al., 2010, 2012). We thus asked whether PSCs preferentially increase at NMJs with age‐related features using S100β‐GFP;thy1‐CFP mice. In these mice, SCs are labeled with GFP and motor axons with CFP (Figure 3a–c and Figure S4a–c). As previously shown (Taetzsch & Valdez, 2018), we observed significant increases in the number of postsynaptic nAChR fragments, blebs, axonal sprouts, and number of polyinnervated NMJs in both the EDL (Figure 3d) and soleus (Figure 3f) of aging S100β‐GFP;thy1‐CFP mice. We further showed that the percentage of fBTX‐labeled receptor area without an innervating axon increased in old EDL muscles (Figure S5). These analyses again reveal that NMJs are affected by aging earlier and more severely in the EDL compared to the soleus.

To determine the relationship between the number of PSCs and age‐related changes at NMJs, we pooled NMJs from middle‐aged and old mice and then separated them by whether they have or lack an age‐related phenotype. We found that PSCs are more abundant at NMJs exhibiting fragmentation, blebbing, and polyinnervation compared to NMJs lacking an age‐induced phenotype in both the EDL (Figure 3e) and soleus muscle (Figure 3g). We also found that PSCs are more abundant at NMJs with axonal sprouts in the EDL muscle (Figure 3e). This was not the case in the soleus where we found a similar number of PSCs in NMJs with and without axonal sprouts (Figure 3g). Together, these data show that age‐related changes at NMJs consistently occur at an earlier age in the EDL compared to the soleus muscles, and that there is a relationship between age‐related abnormalities of the NMJ and number of PSCs.

### 3.5 PSCs form processes during aging that precede axonal sprouts

**FIGURE 4:**
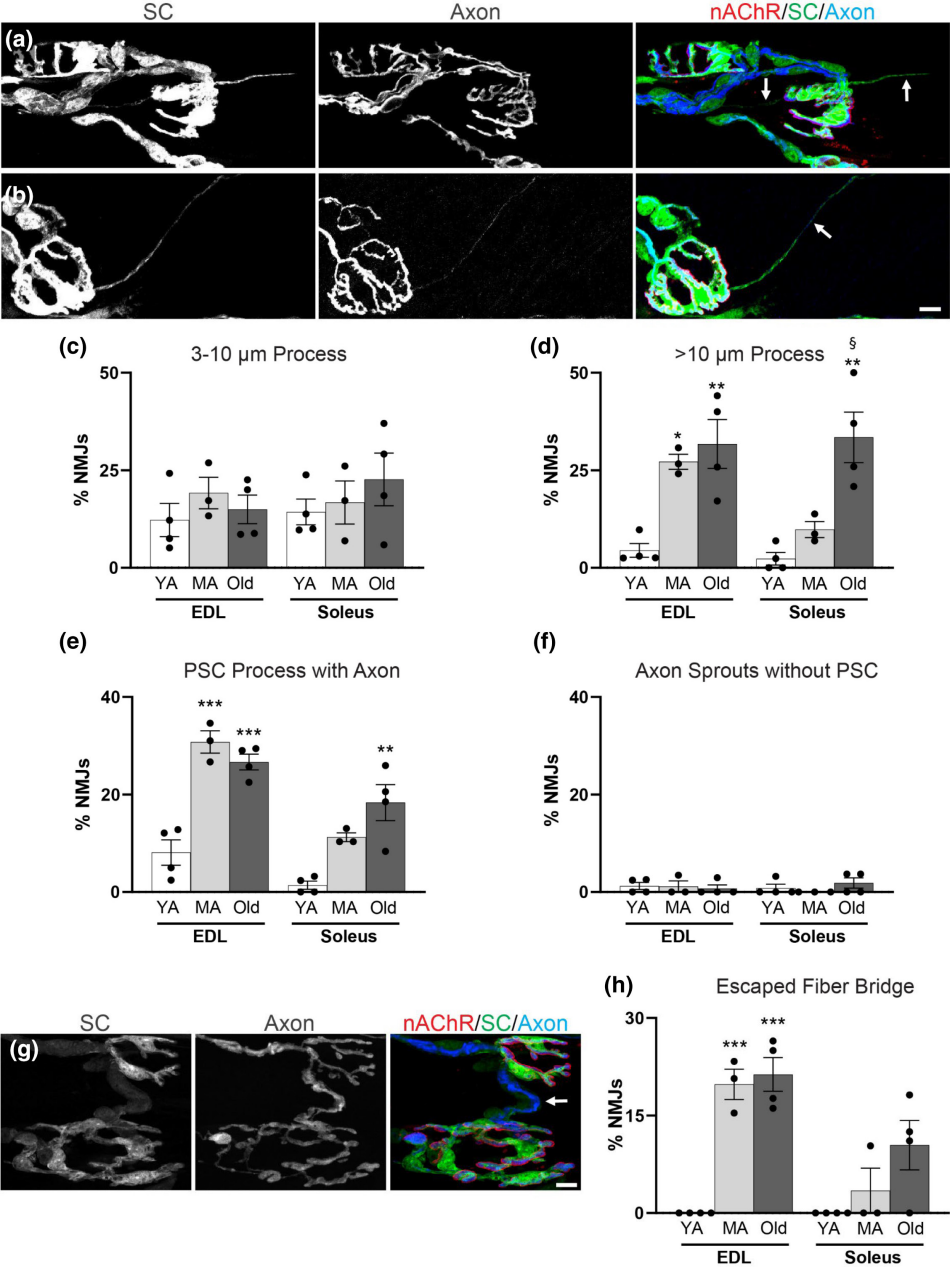
Increased PSC processes and escaped fiber bridges are present at aged NMJs. (a,b) S100β‐GFP;thy1‐CFP EDL NMJs in which PSCs are labeled with GFP (green), motor axons are labeled with CFP (blue) and nAChRs are labeled with fBTX (red). (a) Representative image of a middle‐aged EDL NMJ extending PSC processes lacking axonal sprouts (arrows). (b) Representative image of a middle‐aged EDL NMJ extending a PSC process containing an axonal sprout (arrow). Channel separated, wider‐cropped images with DAPI counterstain available in Figure S6 (a,b). Average percentage of NMJs with 3–10 μm PSC processes (c) and > 10 μm PSC processes (d) in young adult (YA), middle aged (MA) and old EDL and soleus. (e,f) Percentage of NMJs with PSC processes associated with an axon terminal sprout (e) and percentage of NMJs with axon terminal sprouts that are not associated with a PSC process (f) in young adult (YA), middle aged (MA) and old EDL and soleus. (g) Representative image of an escaped fiber bridge in middle‐aged S100β‐GFP;thy1‐CFP EDL. Channel separated, wider‐cropped images with DAPI counterstain available in Figure S6 (d). Percentage of NMJs with escaped fiber bridges in young adult (YA), middle aged (MA) and old EDL and soleus. *p < 0.05, **p < 0.01, ***p < 0.001 versus young adult. ^§^ *p* < 0.05 versus middle aged. One way ANOVA with Bonferroni post hoc. Values represented as mean ± SEM; *n* = 3–4. Scale bar = 10 μm.

Sprouting of the axon terminal, which is an important regenerative function to restore synaptic contact on denervated NMJs, has been previously identified in aging muscle tissue (Li et al., 2011; Valdez et al., 2010, 2012). Axonal sprouting leads to polyinnervation of NMJs, which can degrade the fidelity of motor commands due to muscle fibers receiving information from at least two instead of only one motor neuron (Valdez et al., 2012). On the contrary, axonal sprouting leads to reinnervation of muscle fibers denervated during aging, referred to as “compensatory reinnervation,” which is critical for attenuating muscle atrophy and loss of motor function (Luff, 1998; Soendenbroe et al., 2021; Vandervoort, 2002). Given that axonal sprouts are guided by PSC processes following nerve injury (Son & Thompson, 1995a, 1995b), we sought to determine if PSCs also form processes during aging and whether they are associated with axonal sprouts. To do so, we measured GFP+ and/or CFP+ extensions away from the nAChRs. We defined a PSC process as an extension 3 μm or longer in length extending away from the NMJ (Figure 4a and Figure S6a). It was common to find NMJs with PSC processes without an accompanying axonal sprout between 3 and 10 μm in young adult EDL and soleus muscle, and the incidence of such processes did not change with age in either muscle (Figure 4c). PSCs with processes lacking an axonal sprout greater than 10 μm were rare in young adult muscles. However, the frequency of NMJs with PSC processes greater than 10 μm increased with age in both muscles, although sooner in the EDL (Figure 4D). We next sought to determine the relationship between PSC processes and motor axon sprouts. PSC processes associated with axonal sprouts occurred in young adult mice, though they were not common (Figure S6c). With increasing age, however, the percent of NMJs with PSC processes associated with axonal sprouts (Figure 4b and Figure S6b) significantly increased in both the EDL and soleus muscles (Figure 4e). Though we frequently observed PSC processes without axonal sprouts, we seldom found an axonal sprout without a PSC process at any age (Figure 4f). These data suggest that PSCs form processes prior to motor axons elaborating sprouts.

Following nerve injury, PSC processes extend from one NMJ to another, creating a “bridge” through which an axonal sprout can grow (Kang et al., 2019; Son & Thompson, 1995a, 1995b). Previous investigations of the physiology of aged muscles have suggested that an axonal sprout connected to another NMJ by a PSC process, called an “escaped fiber bridge,” is a common feature of aging muscle (Gordon et al., 2004). We therefore explored the extent to which the phenomenon of escaped fiber bridges exists at the morphological level (Figure 4g and Figure S6d). Escaped fiber bridges were not found in young adult EDL or soleus muscles. During aging, however, many NMJs became associated with an escaped fiber bridge in both muscles (Figure 4h). These data show that PSCs contribute to compensatory reinnervation by forming processes to increase the number of escaped fiber bridges during aging. Through this cellular change, PSCs likely contribute to the increased incidence of polyinnervated NMJs and larger motor unit size in aged muscles.

### 3.6 Electron microscopy reveals process abnormalities and axon engulfment by aged PSCs

**FIGURE 5:**
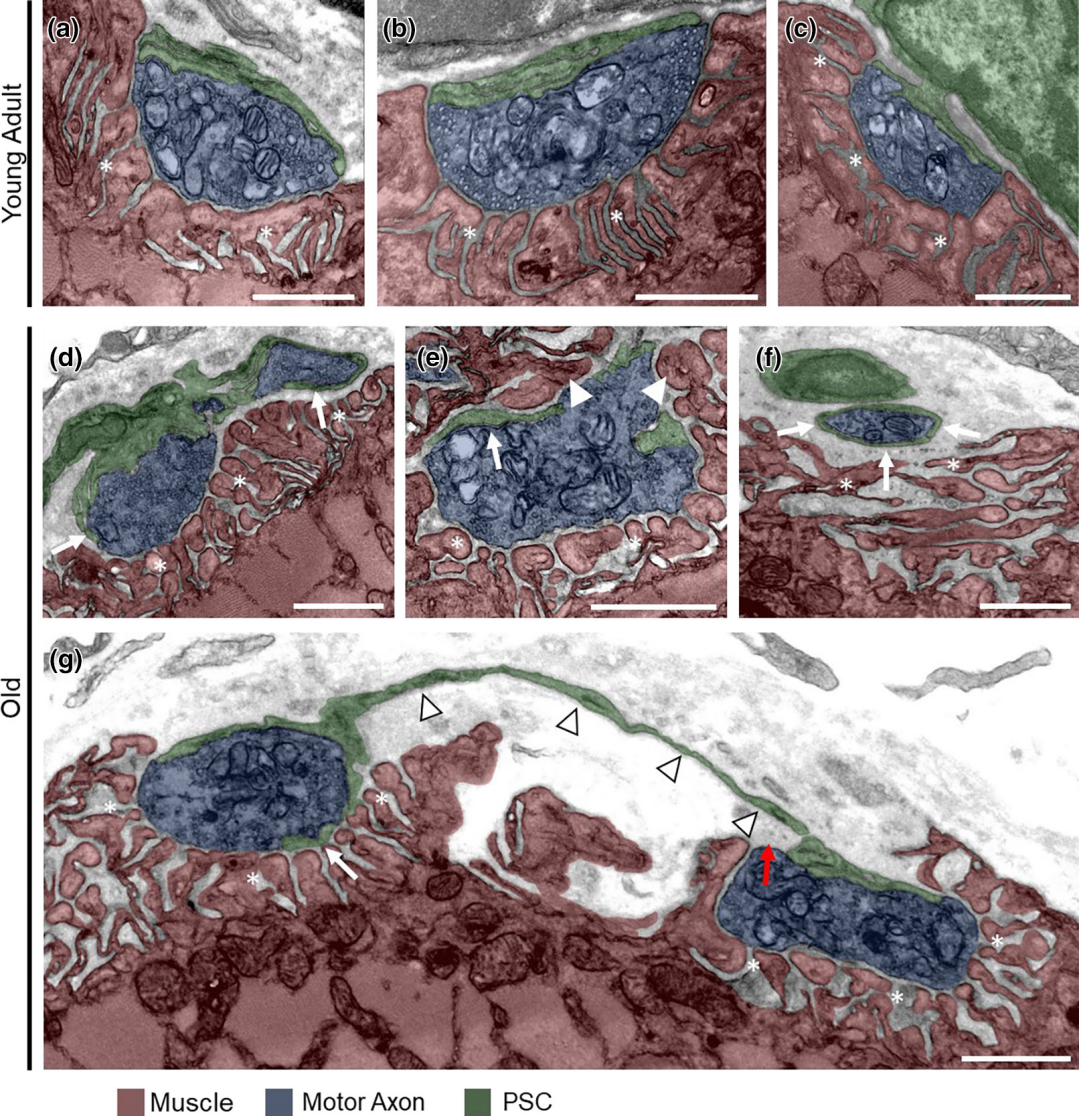
Aged PSCs visualized by electron microscopy. (a‐c) Representative images of young adult NMJs. (d) Old NMJ in which PSC processes (arrows) are extending into the synaptic cleft. (e) Old NMJ in which muscle (arrowhead) mostly surrounds the presynapse, and PSC processes extend between the muscle and presynapse (arrow). (f) Old NMJ in which a PSC process (arrows) surrounds an axon terminal that is retracting from the postsynapse. (g) Old NMJ in which a PSC sprout (arrowheads) connects two presynaptic regions, PSC processes extend between the muscle and presynapse (white arrow), and part of the rightmost part of the presynapse is not fully capped by the PSC (red arrow). Asterisks indicate postsynaptic area. Scale bars = 1 μm.

We used transmission electron microscopy (EM) to better discern the relationship between aged PSCs and NMJs. We examined NMJs in the EDL muscle of 3‐ and 29‐month‐old wild‐type mice. In the young adult mouse, we examined 17 images of NMJs and most exhibited the expected architectural features that include PSCs completely capping the presynapse (Figure 5a–c and Figure S7). By contrast, PSCs and the NMJ exhibited several morphological abnormalities in the old mouse, in which we examined 13 images of NMJs. Aged PSCs only partially capped the presynapse (Young adult = 1 of 17; Old = 6 of 13) (Figure 5g) and processes were instead found either intruding into the synaptic cleft (Young adult = 1 of 17; Old = 12 of 13) or extending to reach another synaptic region (Young adult = 0 of 17; Old = 1 of 13) (Figure 5g). Aged PSCs also partially or fully engulf pieces of axon (Young adult = 0 of 17; Old = 4 of 13), suggesting that aged PSCs may be more phagocytic (Figure 5d,f). Other abnormalities observed at the aged NMJs include disorganized secondary folds, fragmentation of the postsynaptic membrane, and the plunging of the axon terminal along with PSC processes into the myofiber (Figure 5e,f, Figures S8a,b and S9a,b). These data corroborate our light microscopy analysis and published findings (Boaro et al., 1998; Chai et al., 2011; Kawabuchi et al., 2001; Wokke et al., 1990) demonstrating that PSCs and NMJs acquire deleterious structural features during aging.

### 3.7 Migrating SCs contact aged NMJs

During the course of analyzing S100β‐GFP positive PSCs, we found GFP‐positive cells residing outside of, yet contacting, NMJs via processes that resemble those formed by PSCs (Figure S10a). These cells resemble a population of SCs that appear during regeneration of injured nerves (Kang et al., 2019; Reynolds & Woolf, 1992), though it is unclear whether the population we observed in aged tissue is the result of a similar phenomenon. Here, we use the term “migrating Schwann cells (SCs)” to describe SCs that have cell bodies which clearly reside outside the NMJ, with one process contiguous with the NMJ and one process leading away from the NMJ, in contrast with PSCs, which have somata that lie directly over the acetylcholine receptors. In the EDL, these migrating SCs were rare in young adults but then significantly increased during aging, plateauing by middle‐age (Figure S10b). In the soleus, migrating SCs were also rare in young adults but progressively increased during aging (Figure S10b). Migrating SCs resided at a similar distance from the end plate in both EDL (29.1 ± 3.9 μm) and soleus (30.5 ± 5.3 μm) muscles and ranged from a couple of microns away from the NMJ to over 175 microns away.

### 3.8 Molecular mechanisms disrupted in aged PSCs

We profiled the transcriptome of PSCs and all other muscle‐resident SCs using RNA sequencing to identify candidates with roles in PSCs aging. We used fluorescence‐activated cell sorting (FACS) to isolate PSCs and other SCs from S100β‐GFP;NG2‐DsRed mice. In these transgenic mice, PSCs are specifically labeled by the combined expression of GFP and DsRed while other SCs are only labeled with GFP (Castro et al., 2020). We collected PSCs and other SCs from hindlimb muscles of young adult (4–5 months) and old (23–24 months) female mice and performed bulk RNA‐seq. As expected, we found that PSCs have a unique transcriptional signature compared to other SCs (i.e., S100β^+^;NG2^−^ cells) in both young adult and aged mice (Figure S11a,c). These include elevated expression of S100β and NG2 (aka Cspg4) as well as other previously identified markers of PSCs (Castro et al., 2020) in young and old PSCs compared to age‐matched SCs (Figure S11b,d).

**FIGURE 6:**
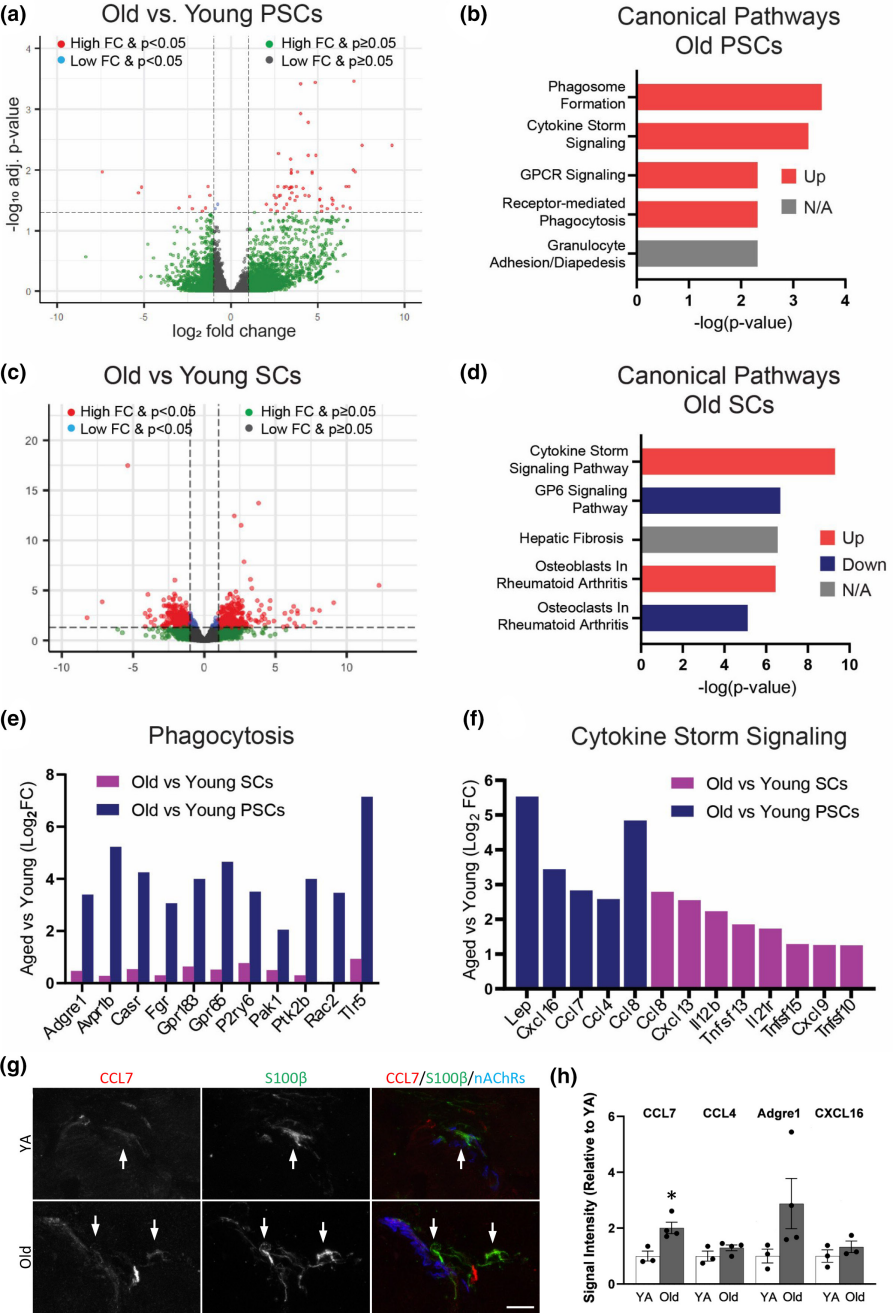
Transcriptomic analysis of aged Schwann cells and PSCs. (a) Volcano plot of gene expression changes in PSCs isolated from skeletal muscles of old versus young adult S100β‐GFP;NG2‐DsRed mice. (b) Top upregulated canonical pathways in old versus young adult PSCs. (c) Volcano plot of gene expression changes in other Schwann cells isolated from skeletal muscles of old versus young adult S100β‐GFP;NG2‐DsRed mice. (d) Top upregulated canonical pathways in old versus young adult Schwann cells. (e) Analysis of changes in expression of genes involved in phagocytosis in old SCs or PSCs, as compared to their young adult counterparts. (f) Cytokine signaling genes that encode secreted signaling factors, as identified by IPA, that are significantly upregulated in old PSCs or SCs, as compared to their young counterparts. (g) Representative images of CCL7 and S100β staining colocalization (arrows) in TA cross sections collected from young adult and old wild type mice. S100β^+^ PSCs were identified by their proximity to fBTX‐labeled nAChRs. (h) Quantification of CCL7, CCL4, Adgre1, and CXCL16 fluorescence intensity in S100β‐labeled PSCs obtained from IHC of TA cross sections collected from young adult and old wild type mice. *p < 0.05 versus young adult, unpaired, 2‐sided *t* test. Values represented as mean ± SEM; *n* = 3–4. Scale bar = 10 μm.

Our comparison of the transcriptional profiles of young versus aged PSCs revealed over 70 differentially expressed genes (DEGs) in aged PSCs (Figure 6a). Aged PSCs presented with changes in canonical pathways related to phagocytosis and cytokine signaling, based on ingenuity pathway analysis (IPA) (Figure 6b). They also exhibited alterations underlying cellular functions such as motility, intercellular signaling, cell death, and survival (Figure S12a). However, aged PSCs did not exhibit alterations in genes with roles in synapse formation and function (Figure S12b). Examination of the impact of aging on the transcriptional composition of other SCs showed over 936 DEGs in aged compared to young SCs (Figure 6c). This included DEGs that have been previously shown (Painter et al., 2014; Verdier et al., 2012) to be altered in aged SCs isolated from sciatic nerves (Table S1). Ingenuity pathway analysis showed that cytokine signaling and cell signaling are among top upregulated canonical pathways (Figure 6d) and cellular functions (Figure S12c), respectively, in old compared with young SCs.

We next asked whether DEGs and pathways in aged PSCs differed from aged SCs. Genes related to phagocytosis were found to be altered only in aged PSCs (Figure 6e). Aged PSCs presented with elevated expression of *Casr*, *Adgre1*, *Tlr5*, and other genes that mediate phagocytosis in macrophages. Genes suspected or shown to mediate SC phagocytosis (Lutz et al., 2017) were not statistically altered in aged PSCs (Figure S12d). However, it is worth noting that aged PSCs express lower levels of *megf10* (Log_2_ FC = −1.12), which is highly enriched in young and aged PSCs compared to other SCs, and is a well‐known modulator of orderly spatial distribution and phagocytic activity of glial cells in the CNS (Chung et al., 2013; Kay et al., 2012). These findings suggest that phagocytosis may be a function that is uniquely altered in aged PSCs among all SCs. Additionally, this analysis revealed that aged PSCs and SCs upregulate different genes related to cytokine signaling (Figure 6f and Figure S12e). We also examined aged PSCs and SCs for changes in genes differentially expressed by SCs following nerve injury and with roles in nerve repair and axonal regeneration (Jessen & Mirsky, 2016; Ma et al., 2018). We found that the majority of these genes were not differentially expressed in either aged PSCs or SCs (Table S2). We then utilized immunohistochemistry to examine protein levels for four genes with increased transcript number in aged PSCs: Ccl7, Ccl4, Adgre1 (F4/80), and Cxcl16. Intensity profiles of the stains for these proteins were compared to the intensity of S100β staining, for which transcript number remain unchanged in old compared with young PSCs, at NMJs of young adult and old TA muscles. Ccl7 protein levels indicated a significant increase in old compared with young PSCs (Figure 6g and Figure 6h), though it is possible that other cells such as macrophages also contribute to the increased levels of Ccl7 at and near the NMJ. Furthermore, this analysis revealed a possible trend towards increased expression, though not statistically significant, of Adgre1 (Figure 6h and Figure S13). Together, these results suggest that both aged PSCs and SCs upregulate genes related to cytokine signaling and adopt a phenotype geared towards recruiting immune cells and repairing axonal damage, as has been reported previously following nerve injury (Jessen & Mirsky, 2016). PSCs are unique; however, in that they upregulate a number of well‐described regulators of phagocytosis which suggest that aged PSCs are increasingly responsible for removing cell debris from the NMJ with age.

## 4 DISCUSSION

The aim of this study was to define the impact of aging on PSCs. We discovered that PSCs are more abundant yet fail to completely cover the presynaptic region of aged NMJs. PSCs also form processes, with some reaching adjacent NMJs in aged mice. We show that while many of these PSC processes were associated with axonal sprouts, axonal sprouts were almost never found without a PSC process. Additional cellular analysis revealed PSCs intruding into the synaptic cleft, losing their organized spatial distribution, and possibly phagocytosing the presynapse. Molecular analysis supported our cellular observations by revealing genes and pathways with potential roles in PSCs and NMJ aging. We found genes and pathways involved in phagocytosis, inflammation, and intercellular signaling altered in aged PSCs. These findings provide insights for the first time about the cellular and molecular mechanisms disrupted in aged PSCs with roles in NMJ degeneration.

### 4.1 PSC increase during aging possibly due to migration of other SCs onto the NMJ

We deployed approaches to indelibly mark and thus faithfully determine the number of PSCs at NMJs in aging mice. We found that PSCs remain at NMJs, and in many cases increase in number, during aging in stark contrast to published studies (Ikemoto‐Uezumi et al., 2022; Snyder‐Warwick et al., 2018). In addition to our thorough analysis of EDL and soleus NMJs, we also surveyed old sternomastoid (STM) muscles with both S100β immunostaining and the S100β‐GFP transgene, and found that PSCs remain at old NMJs (Figure S14), consistent with published research that examined old STM NMJs but did not report any absence of PSCs (Li et al., 2011). We found that PSCs are more abundant at aged NMJs, particularly those exhibiting fragmentation, blebbing, and polyneuronal innervation in both the EDL and soleus muscles. This finding is in line with published studies showing that the number of PSCs changes at NMJs undergoing dramatic structural changes during development (Castro et al., 2020; Darabid et al., 2014), recovery from various injuries (Hastings et al., 2020; Love & Thompson, 1998), and muscle diseases (Haddix et al., 2018; Lee et al., 2011).

Our data showing an increase in PSC number with age naturally raises the question: what is the origin of new PSCs in adulthood? In addition to more PSCs at the NMJ, we also found other SCs located outside of, but clearly contacting, NMJs in aged mice. These SCs did not appear to emanate from an adjacent NMJ, nor from SCs located along the innervating axon prior to it reaching the NMJ, yet shared common features with PSCs, expressing both GFP and DsRed in aged S100β‐GFP;NG2‐DsRed mice (Figure S1A). Furthermore, we found no Ki67+ nuclei, a marker for proliferating cells, at the NMJ in either young or old TA muscle, indicating a lack of local proliferating cells at the synapse (Figure S15). Based on these findings, we propose that a unique SC progenitor subtype, which migrates onto NMJs and differentiates into PSCs after contact with the NMJ, may be responsible for the increased number of PSCs at aged NMJs. However, it is plausible that migrating SCs may instead be PSCs leaving the NMJ and that PSCs undergo intermittent bouts of proliferation during aging which our analysis failed to capture. Thus, more in‐depth investigation will be required to determine where new PSCs come from in adult mammals.

### 4.2 The rate of PSC aging varies among muscles with different susceptibility to aging

Our study revealed notable differences between PSCs in the EDL, soleus, and EOMs. PSCs and NMJs are affected by aging earlier in the EDL compared to the soleus muscles. Additionally, the number of PSCs increased without a concomitant enlargement of the junctional area in the EDL during aging. This was not the case in the soleus where both the number of PSCs and junctional area increased during aging. In EOMs, where NMJs have been shown to resist age‐related changes, the number of PSCs remain unchanged in aged mice. What explains these differences between the EDL, soleus, and EOMs? The most plausible explanation is that intrinsic properties unique to each muscle underpin the differences in the rate of PSC addition to NMJs during aging. These include differences in the myofiber type composition, functional demand, and susceptibility to age‐induced degeneration. It is also plausible that neuregulin 1 signaling or other molecular pathways with key roles in SC proliferation and differentiation as well as NMJ stability may be differentially dysregulated in the EDL and soleus compared to EOMs during aging (Jessen & Mirsky, 2005; Lee et al., 2016; Taveggia, 2016; Trachtenberg & Thompson, 1996). Regardless, these findings have implications for understanding the roles of PSCs at NMJs and muscles with different susceptibility to aging.

### 4.3 Cellular mechanisms disrupted in aged PSCs

A primary role of PSCs is to cap the pre‐ and post‐synaptic regions of the NMJ. This allows PSCs to insulate the synaptic cleft from molecules present elsewhere in muscles and vice versa. PSCs tightly cap the presynapse and also serve as a barrier between the synaptic cleft and the extrasynaptic region of muscles (Darabid et al., 2014; Ko & Robitaille, 2015). Using electron microscopy (EM), we found that aged PSCs fail to adequately cap the presynapse and synaptic cleft. Electron microscopy also revealed that aged PSCs extend processes into the synaptic cleft, which likely blocks important signaling events between the pre‐ and post‐synapse. Furthermore, we found that PSCs form additional processes that project away from the end plate, even in healthy young NMJs, using light microscopy. PSC processes with and without axonal sprouts reach other, already innervated NMJs, in aged mice, a phenomenon that may contribute to the increased incidence of polyinnervated NMJs reported here and in previously published studies. Why would PSCs processes grow towards an already innervated NMJ? We hypothesize that the failure of PSCs to cap aged NMJs leads to the diffusion of synaptic factors that promote the growth of and attract PSC sprouts from nearby NMJs. The impetus for PSC process formation in aged muscle may be the result of the same molecular mechanism that causes the elaboration and extension of PSC processes following denervation (Reynolds & Woolf, 1992; Son & Thompson, 1995a; Son & Thompson, 1995b) and in ALS (Arbour et al., 2015). Together, our data indicate that polyinnervation and compensatory reinnervation of NMJs during aging both result from PSCs forming processes to guide motor axons to both denervated NMJs and those that are incompletely capped by PSCs. Thus, PSC processes may contribute to the increased amplitude of motor units found in old age (Luff, 1998) by increasing the number of muscle fibers a given motor neuron innervates. At the same time, they may contribute to age‐related motor dysfunction by increasing polyinnervation of muscle fibers.

### 4.4 Molecular mechanisms altered in aged PSCs and other SCs in muscles

This study revealed, for the first time, the molecular landscape of aged PSCs as well as other SCs in muscles. By comparing the transcriptome of PSCs and other SCs, we identified genes differentially expressed between PSCs and all other SCs and across ages. We discovered that aged PSCs, but not other SCs, upregulate genes with known roles in mediating macrophage phagocytosis. Given that aging is a chronic state, this functional phenotype of PSCs could be either beneficial or detrimental to NMJ remodeling and stability. The clear benefit of such functional shift is that it would allow PSCs to continuously clear cellular debris, regardless of source, that may be more prevalent at aged NMJs. Support for this idea was provided by work from Kang and Lichtman (Kang & Lichtman, 2013). They found that axonal regeneration is delayed in aged mice due to inefficient removal of axonal debris by SCs that populate the endoneurial tube. But once the axon reaches the NMJ, reinnervation proceeds at a normal rate, indicating that aged PSCs retain their ability to efficiently remove axonal debris (Kang & Lichtman, 2013). Thus, the adoption by PSCs of macrophage‐related phagocytic mechanisms may help them efficiently clear the end plate of debris so motor axons can reach and reconnect with the postsynaptic region of the NMJ (Kang & Lichtman, 2013). However, it is possible that macrophage‐related phagocytic mechanisms may cause PSCs to erroneously target the presynapse, affecting its stability, and consequently contributing to its degeneration.

Aged PSCs and other SCs were also found with altered expression of unique sets of genes implicated in cytokine signaling. Leptin (*Lep*), *Cxcl16*, *Ccl7*, and *Ccl4* were found elevated specifically in aged PSCs. Some of these cytokines have been implicated in modulating synaptic function in normal conditions and are affected by Alzheimer's Disease (Di Castro et al., 2016; Irving & Harvey, 2014). Through IHC analysis, we found that protein levels of Ccl7 were significantly increased in old versus young adult PSCs. Ccl7 is a chemoattractant for immune cells such as macrophages, and therefore may be a signaling pathway through which PSCs participate in inflammation of aged muscle. We also observed a trend towards increased expression of Adgre1 (F4/80) protein in aged PSCs. Adgre1 is a marker for macrophages, and these data suggest that aged PSCs may be acquiring a more macrophage‐like phenotype in old age. In all other SCs, three members of the tumor necrosis factor superfamily and additional cytokines were found to be increased in aged mice. This study also revealed other pro‐inflammatory molecules altered in both PSCs and other SCs. Together, these data provide leads to target specific genes and pathways in PSCs and other SCs to prevent NMJ and axonal degeneration during aging.

## AUTHOR CONTRIBUTIONS

G.V. and R.L.H. were involved in conceptualization, methodology, supervision, validation, writing‐original draft, and writing—reviewing and editing. G.V., M.F.A, and R.L.H. were involved in formal analysis. E.S., D.J., A.O., J.P.D.S., and R.L.H. were involved in investigation. M.F.A, and R.L.H. were involved in data curation and visualization. G.V. was involved in resources, funding acquisition, and project administration.

## FUNDING INFORMATION

This work was funded through grants from the National Institute on Aging (R01AG055545 and R56AG051501) and the National Institute of Neurological Disorders and Stroke (R21NS106313) awarded to GV. RLH was supported in part by NRSA Institutional Research Training Grant T32 AG041688‐11. ES was supported in part by NRSA Institutional Research Training Grant T32 GM136566‐03.

## CONFLICT OF INTEREST STATEMENT

None declared.

### OPEN RESEARCH BADGES

This article has earned Open Data, Open Materials and Preregistered Research Design badges. Data, materials and the preregistered design and analysis plan are available at [[insert provided URL(s) on the Open Research Disclosure Form]].

## Supporting information


Figures S1–S15

Tables S1–S2

Appendix S1

## Data Availability

The data that support the RNA‐seq findings of this study are openly available in NCBI GEO at https://www.ncbi.nlm.nih.gov/geo/, reference number GSE222631. The data that support all other findings of this study are openly available in Harvard Dataverse at https://doi.org/10.7910/DVN/UKTKV5, reference number doi:10.7910/DVN/UKTKV5.
