# Supplementary material for: Cellular and molecular evidence that synaptic Schwann cells contribute to aging of mouse neuromuscular junctions
Source: Aging Cell. 2023 Sep 28;22(11):e13981. doi: 10.1111/acel.13981 (PMC10652323; doi:10.1111/acel.13981)
Supplement: Supplementary file 1 — Figures S1–S15 [file ACEL-22-e13981-s003.pdf]

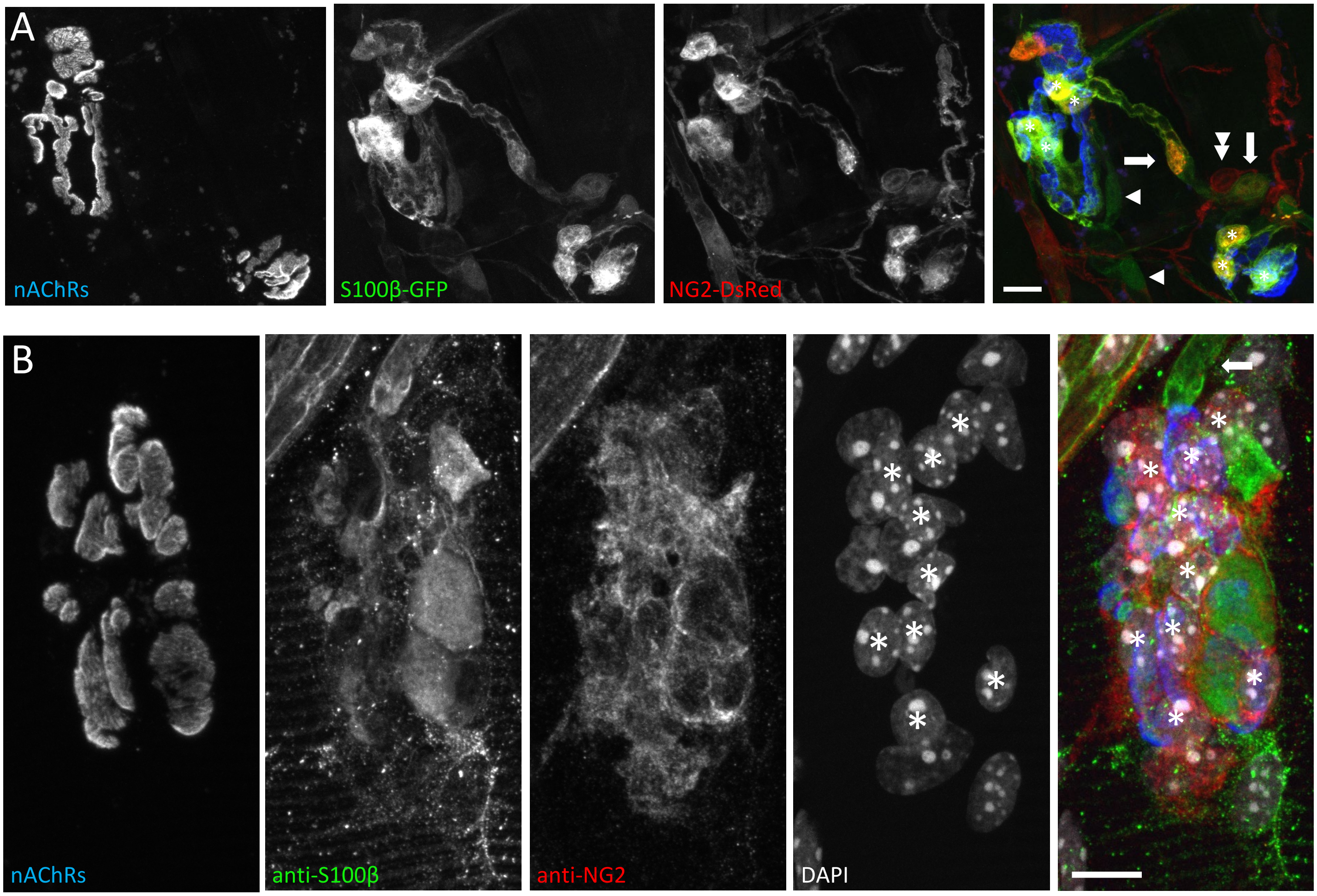

Supplementary Figure 1. PSCs at old NMJs express both S100β and NG2. (A) Soleus NMJs from an old female S100β-GFP;NG2-DsRed mouse. Asterisks indicate PSC cell bodies. Arrows indicate nuclei along an escaped fiber bridge that express both GFP and DsRed. GFP-only (arrowheads) and DsRed-only (double arrowheads) also reside outside of the NMJ region. (B) EDL NMJ from an old female WT mouse immunostained for S100β and NG2. Asterisks indicate PSC cell bodies. Arrow indicates the final myelinating Schwann cell that is positive for S100β, but negative for NG2. Scale bars 10 μm.

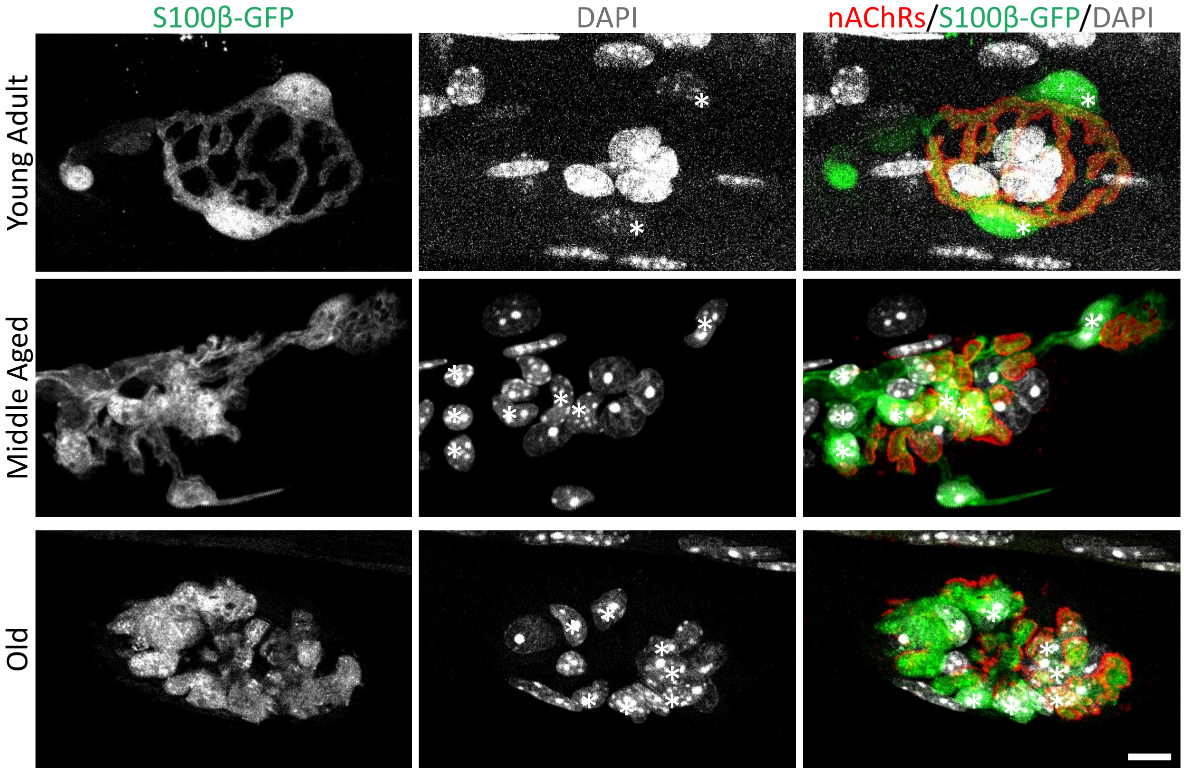

Supplementary Figure 2. Separated S100β-GFP and DAPI channels for images in Figure 1A, merged with nAChRs in final panel. Asterisks indicate PSC cell bodies. Scale bar 10μm.

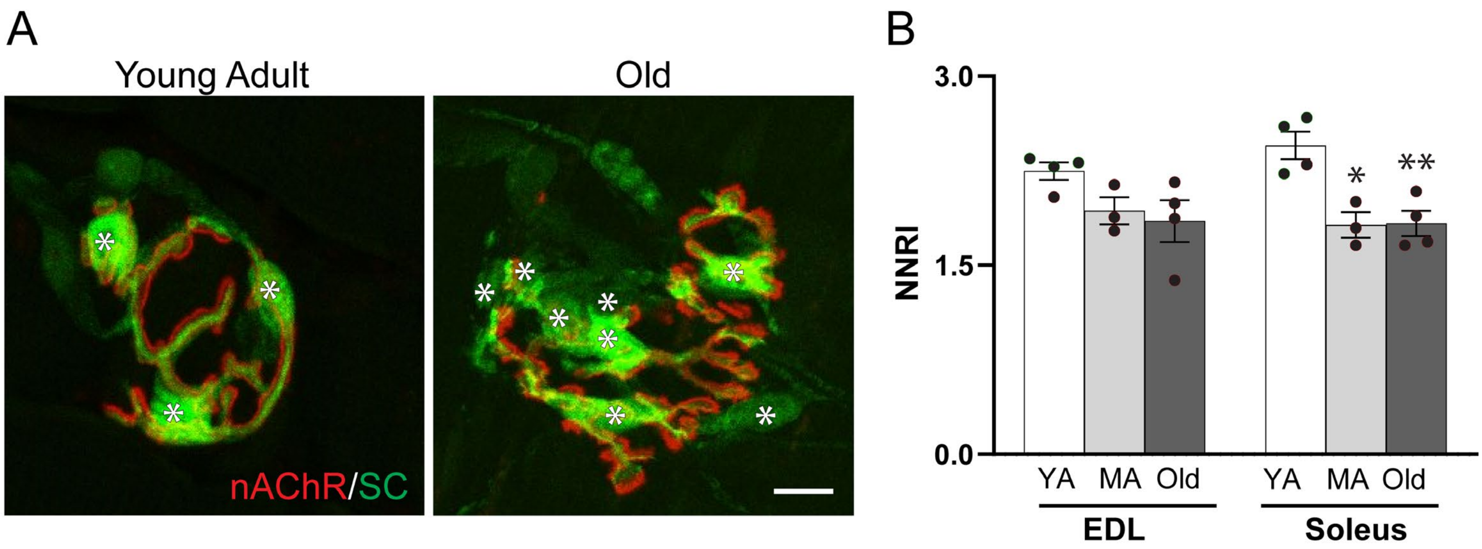

Supplementary Figure 3. (A) Representative images of PSC distribution in soleus of young adult and old S100β-GFP mice in which PSCs are labeled with GFP (green) and nAChRs are labeled with fBTX (red). (B) Average nearest neighbor regularity index (NNRI) values for PSCs in young adult (YA), middle aged (MA) and old EDL and soleus. Values represented as mean NNRI ± SEM. * $p < 0.05$, ** $p < 0.01$ versus young adult, one way ANOVA with Bonferroni post hoc, n = 3-4 mice. Scale bar = 10 μm.

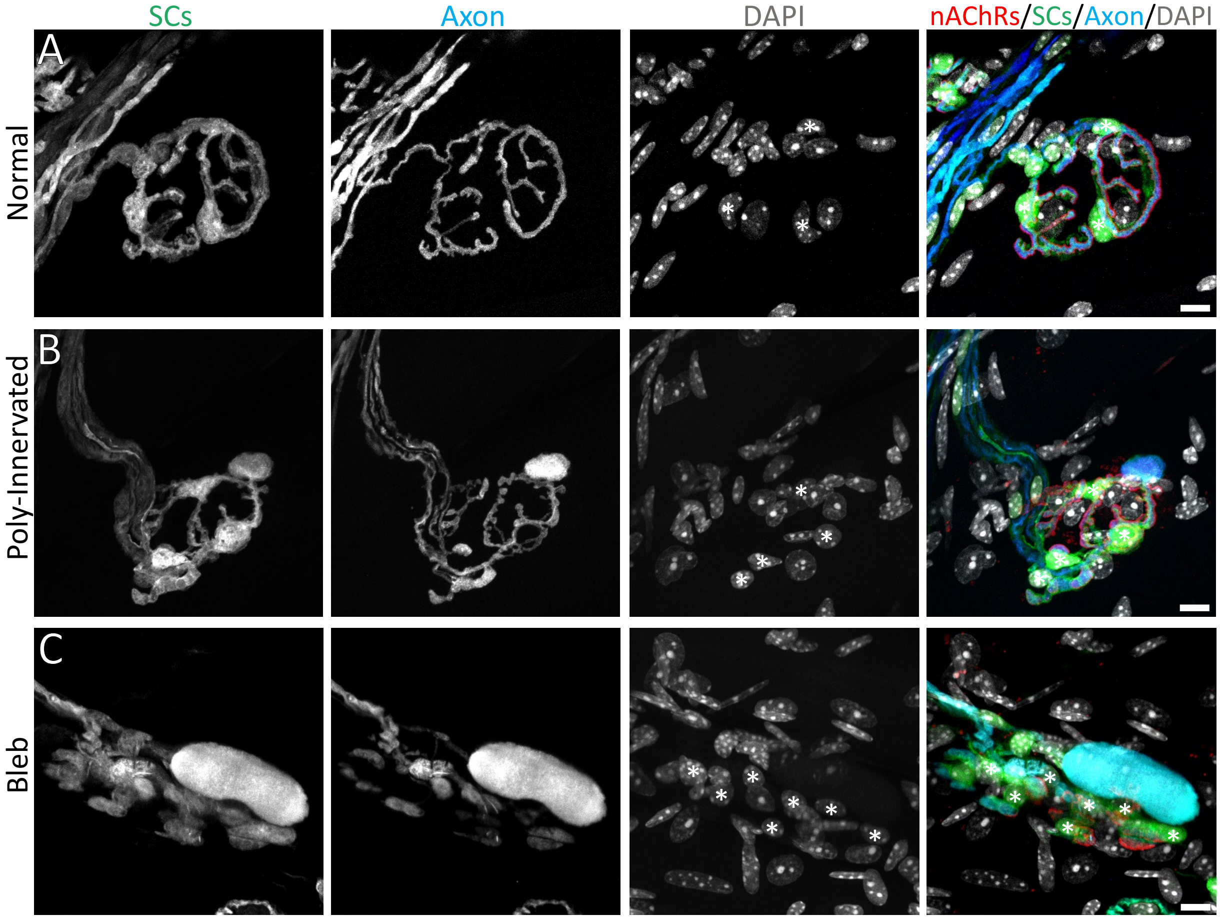

Supplementary Figure 4. Wide-cropped versions of images in Figure 3A-C. S100β-GFP, thy1-CFP, and DAPI channels are separated and merged with nAChRs in final panel. Asterisks indicate PSC cell bodies. Scale bars 10μm.

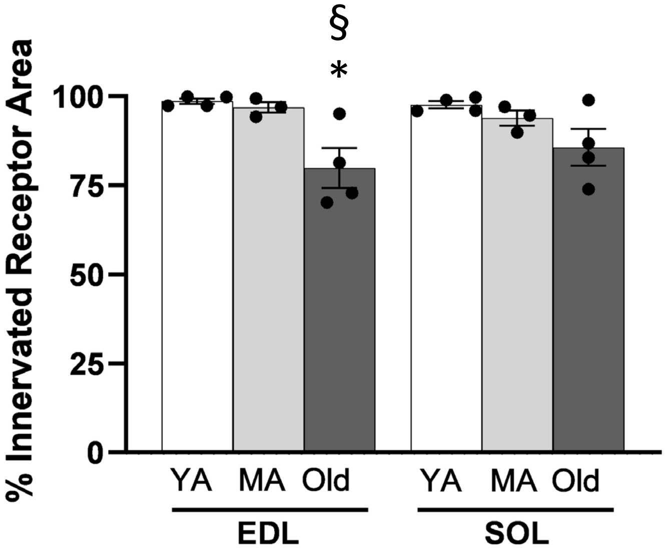

Supplementary Figure 5. Average innervation of nAChRs decreases in old EDLs. Average percentage of nAChR area innervated by CFP+ axon terminal. * $p < 0.05$ versus young adult. § $p < 0.05$ versus middle aged. One way ANOVA with Bonferroni post-hoc. Values represented as mean ± SEM; n = 3-4.

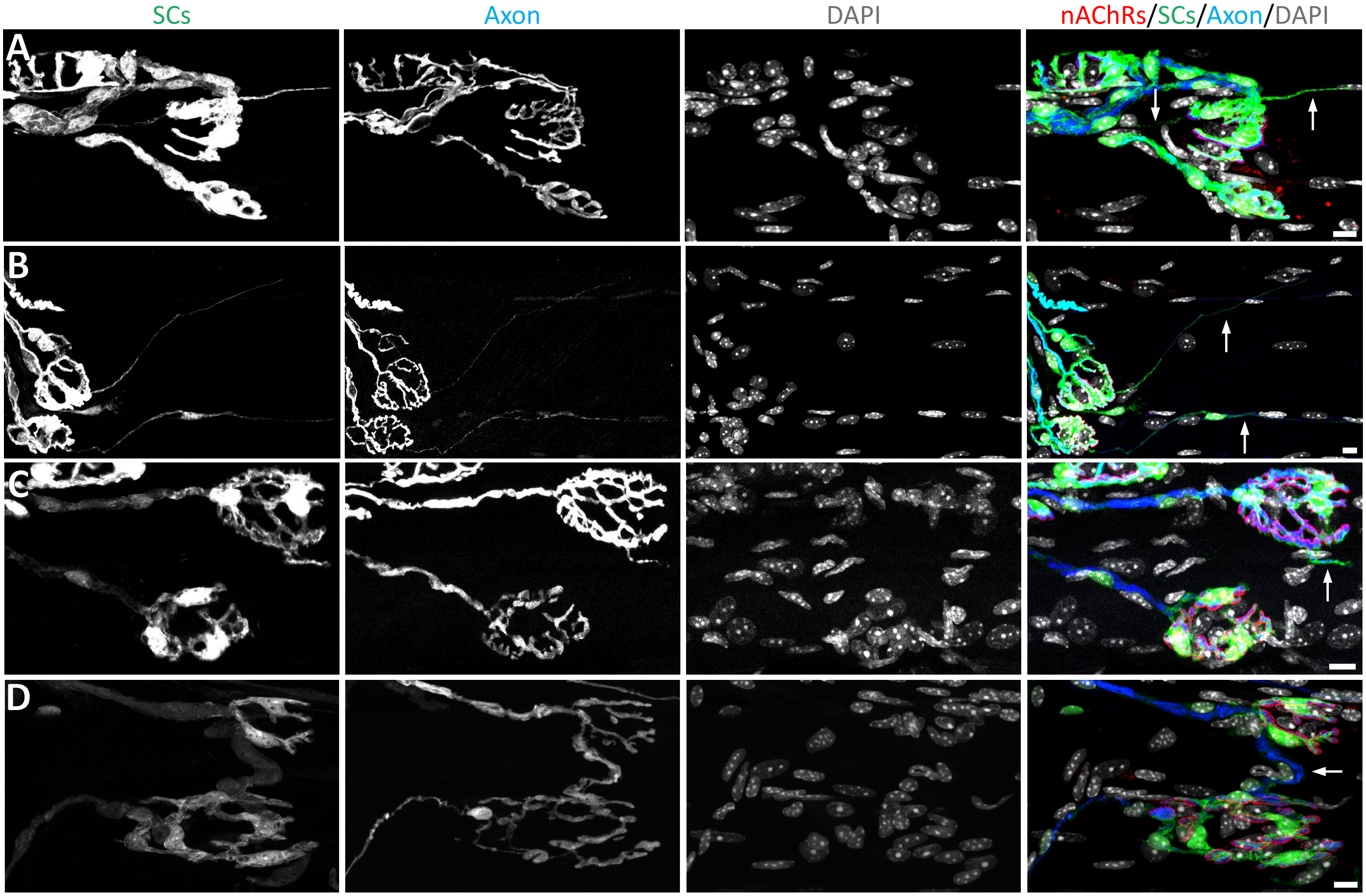

Supplementary Figure 6. Wide-cropped images of PSC processes. S100β-GFP, thy1-CFP, and DAPI channels are separated and merged with nAChRs in final panel. (A,B) Wide crop versions of image in Figure 4A (A) and Figure 4B (B). Arrows indicate PSC processes. (C) Image of a young adult EDL NMJ with a PSC process containing an axonal sprout (arrow). (D) Wide crop version of image in Figure 4G. Arrow indicates escaped fiber bridge. Scale bars = 10 μm.

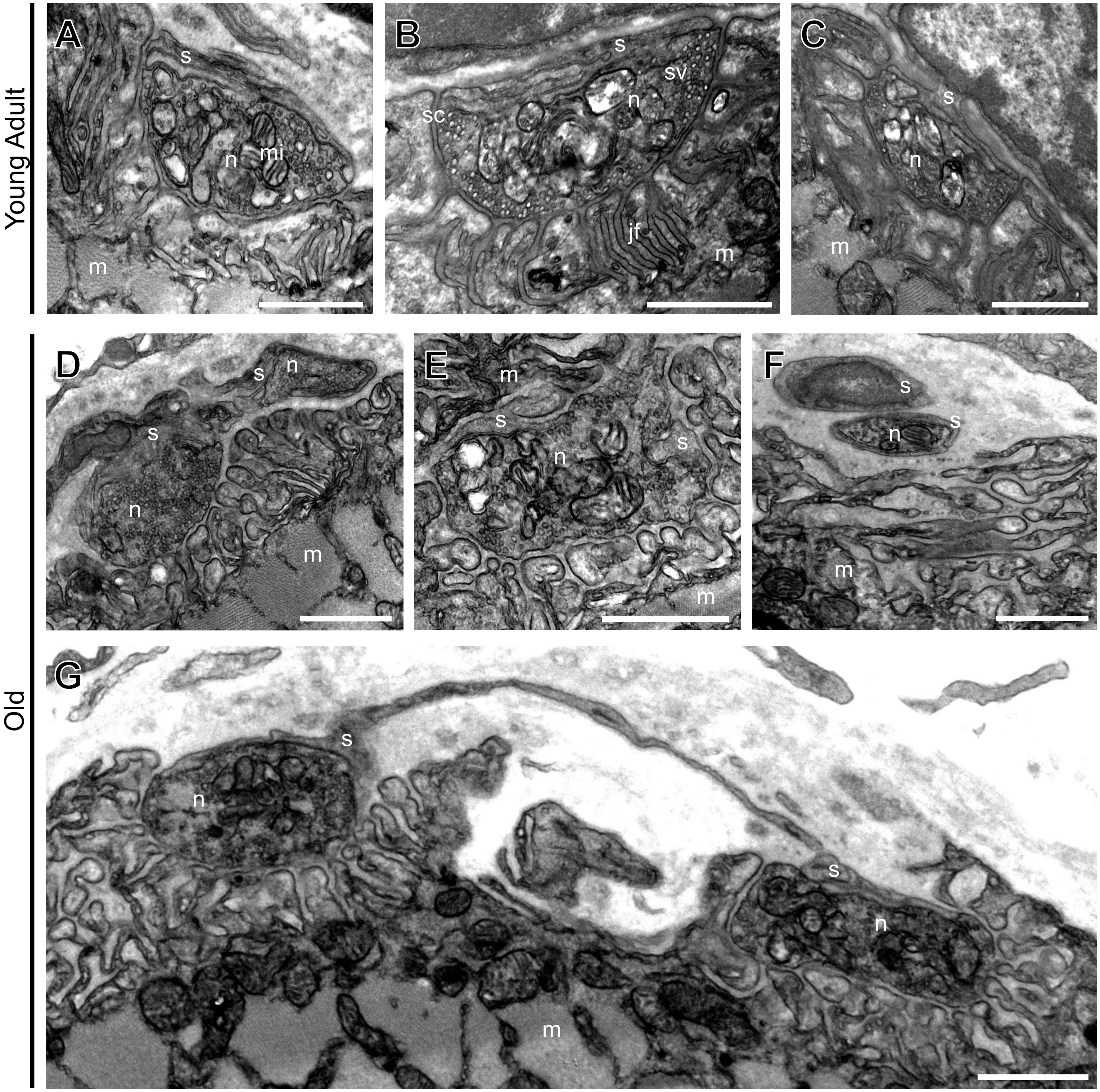

s = perisynaptic Schwann cell, n = nerve terminal, m = muscle,
mi = mitochondria, sc = synaptic cleft, sv = synaptic vesicles, jf = junctional folds

Supplementary Figure 7. Non-pseudo colored version of Figure 5. s = perisynaptic Schwann cell, n = nerve terminal, m = muscle, mi = mitochondria, sc = synaptic cleft, sv = synaptic vesicles, jf = junctional folds. Scale bars = 1 μm.

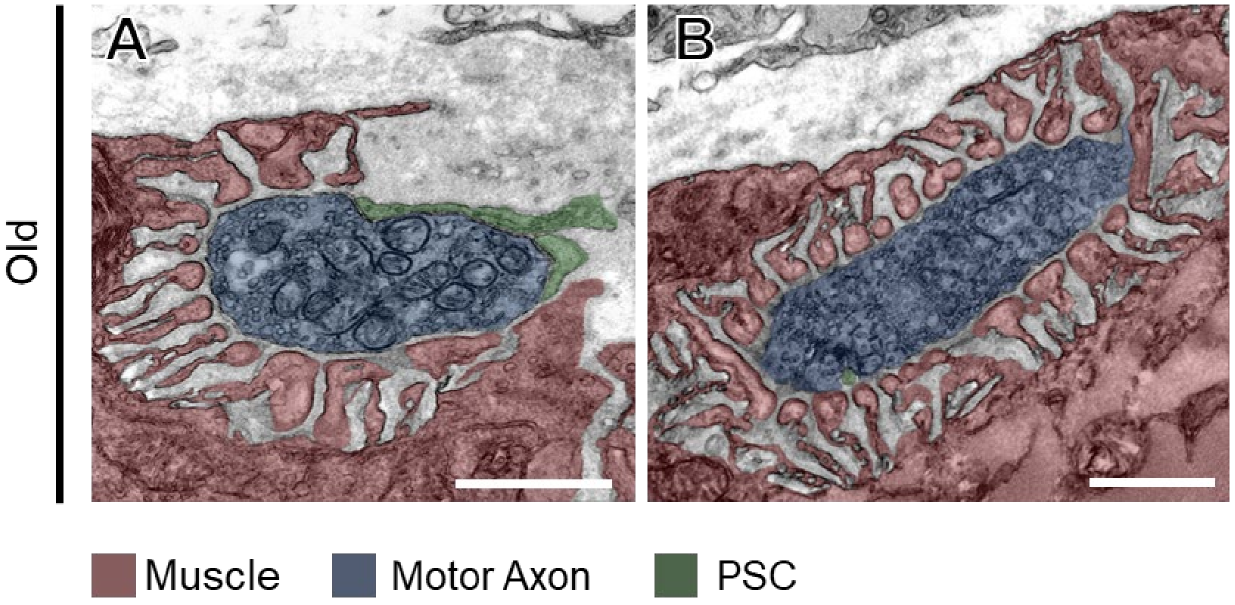

Supplementary Figure 8. (A) Old NMJ in which the axon terminal plunges into the muscle. (B) Old NMJ in which the axon terminal and a small PSC process are completely surrounded by muscle. Scale bars = 1 μm.

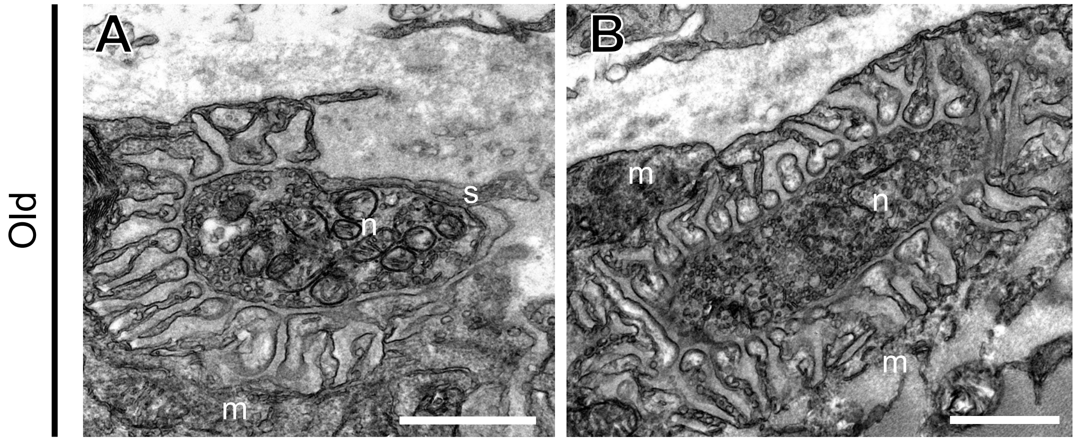

s = perisynaptic Schwann cell, n = nerve terminal, m = muscle

Supplementary Figure 9. Non-pseudo colored version of Supplementary Figure 8. s = perisynaptic Schwann cell, n = nerve terminal, m = muscle. Scale bars = 1 μm.

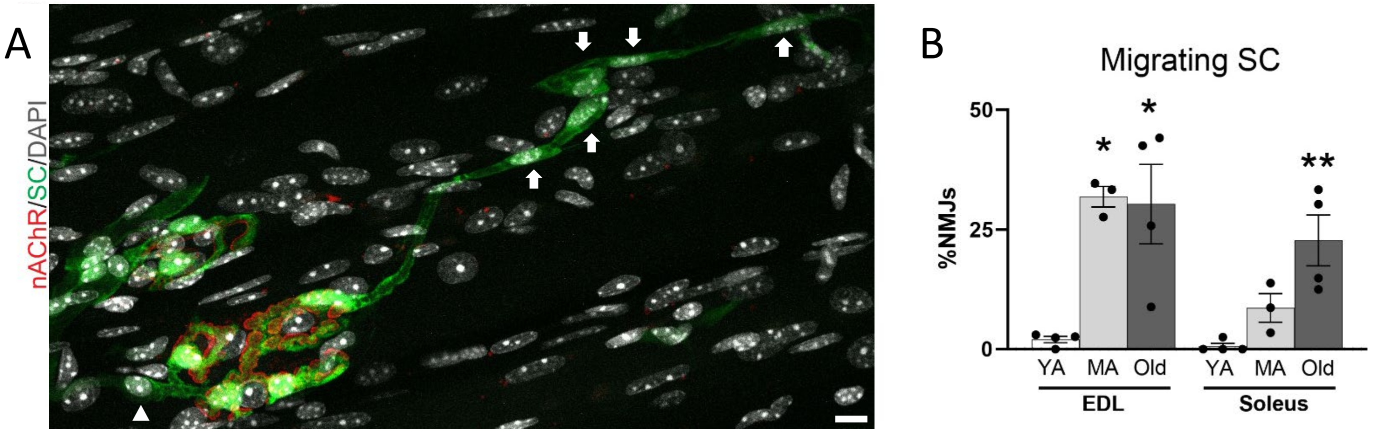

Supplementary Figure 10. Migrating Schwann cells are abundant in old muscles (A) Representative image of GFP-labeled migrating Schwann cells (arrows) in middle aged S100β-GFP EDL. Top NMJ in image is not associated with migrating Schwann cells. Arrowhead denotes myelinating SC associated with axon. (B) Percentage of NMJs with migrating Schwann cells in young adult (YA), middle aged (MA) and old EDL and soleus. * $p < 0.05$, ** $p<0.01$ versus young adult. One way ANOVA with Bonferroni post-hoc. Values represented as mean ± SEM; n = 3-4. Scale bars = 10 μm.

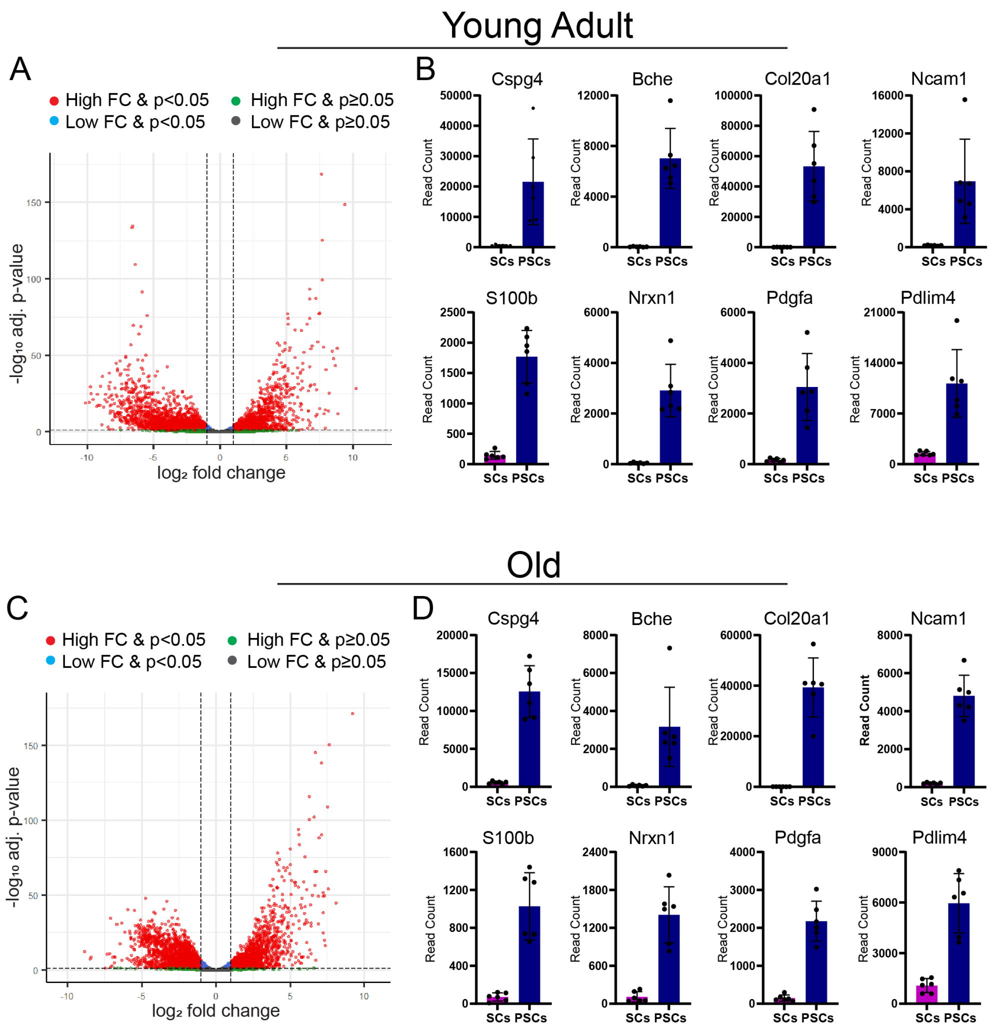

Supplementary Figure 11. (A) Volcano plot of gene expression changes in PSCs versus other Schwann cells isolated from skeletal muscles of young adult S100β-GFP;NG2-DsRed mice. (B) Read counts of genes that have been previously identified to be highly enriched in PSCs versus other Schwann cells in young adult PSCs and Schwann cells. (C) Volcano plot of gene expression changes in PSCs versus other Schwann cells isolated from skeletal muscles of old S100β-GFP;NG2-DsRed mice. (D) Read counts of genes that have been previously identified to be highly enriched in PSCs versus other Schwann cells in old PSCs and Schwann cells.

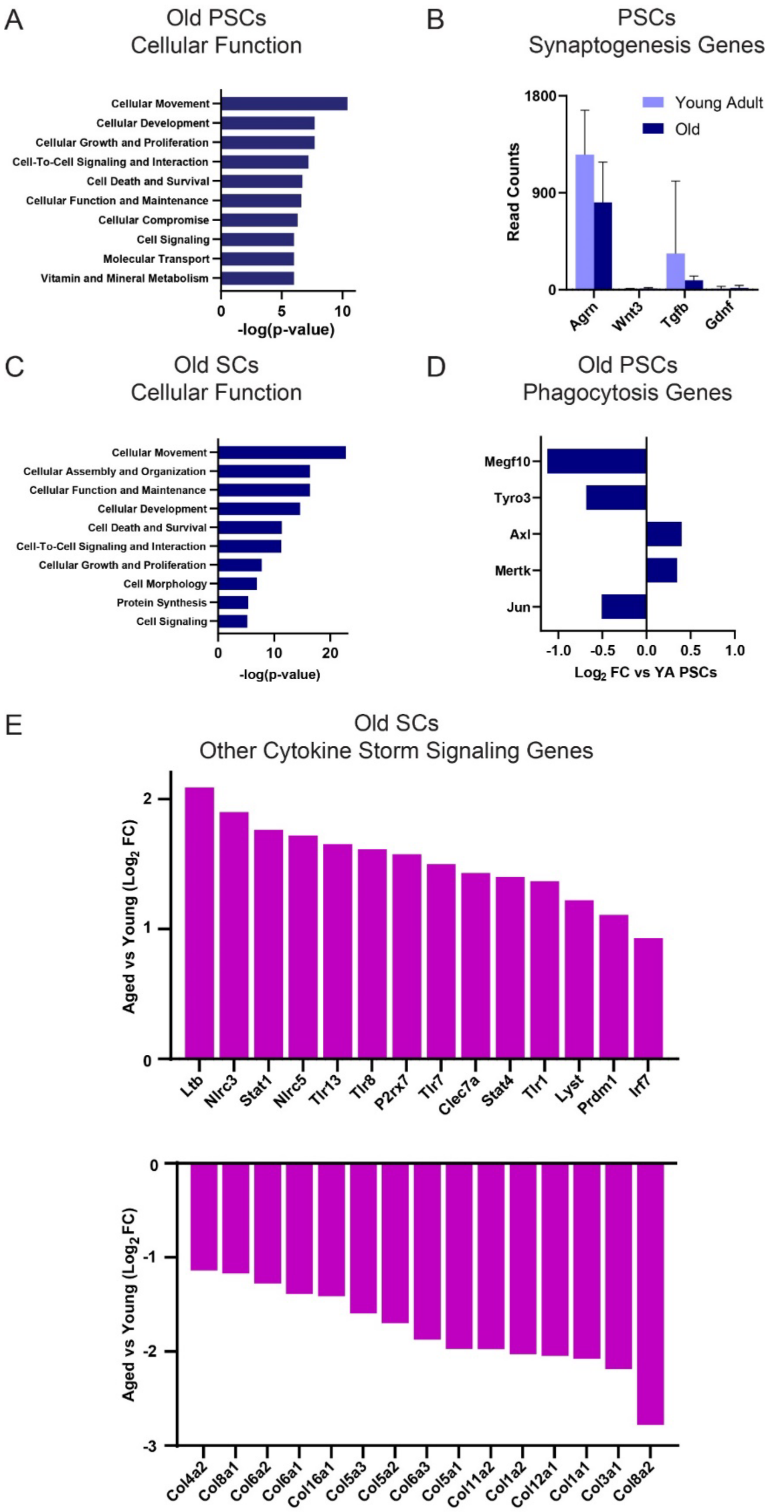

Supplementary Figure 12. (A) Top upregulated cellular functions in old versus young adult PSCs. (B) Read count values of NMJ synaptogenesis genes in young adult and old PSCs. (C) Top upregulated cellular functions in old versus young adult SCs. (D) Log_2 fold change values of other mediators of phagocytosis in old versus young PSCs. (E) Cytokine signaling genes that encode non-secreted factors or collagens, as identified by IPA, that are differentially expressed in old versus young adult SCs.

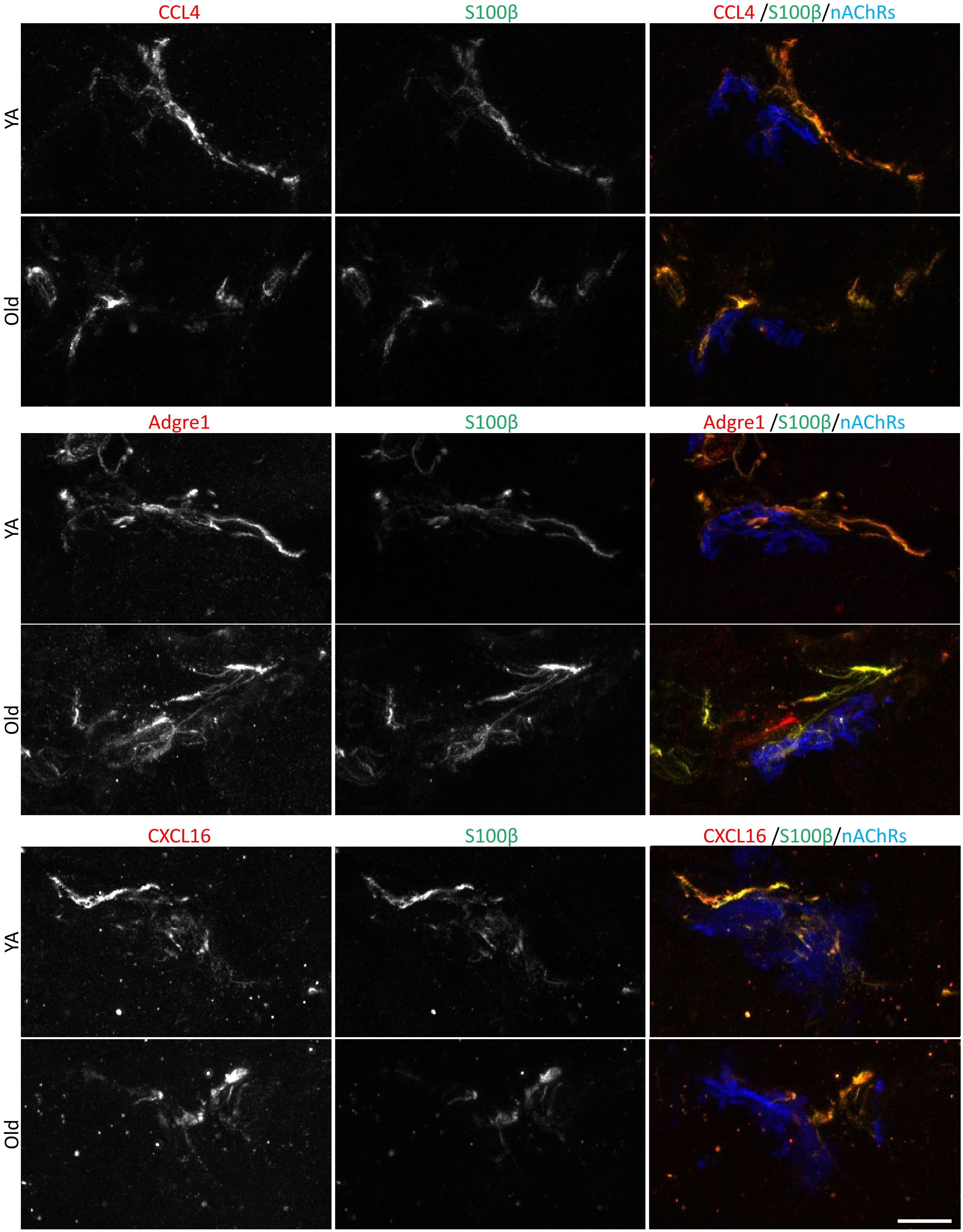

Supplementary Figure 13. IHC for genes with age-related increased transcription. Representative IHC images for three genes (CCL4, Adgre1, and CXCL16) shown to increase transcription in old age via bulk RNA sequencing. NMJs from TA muscles are shown from Young Adult (YA) and Old mice and counterstained with anti-S100β antibody to label SCs and fBTX to label nAChRs. Scale bar = 10 μm.

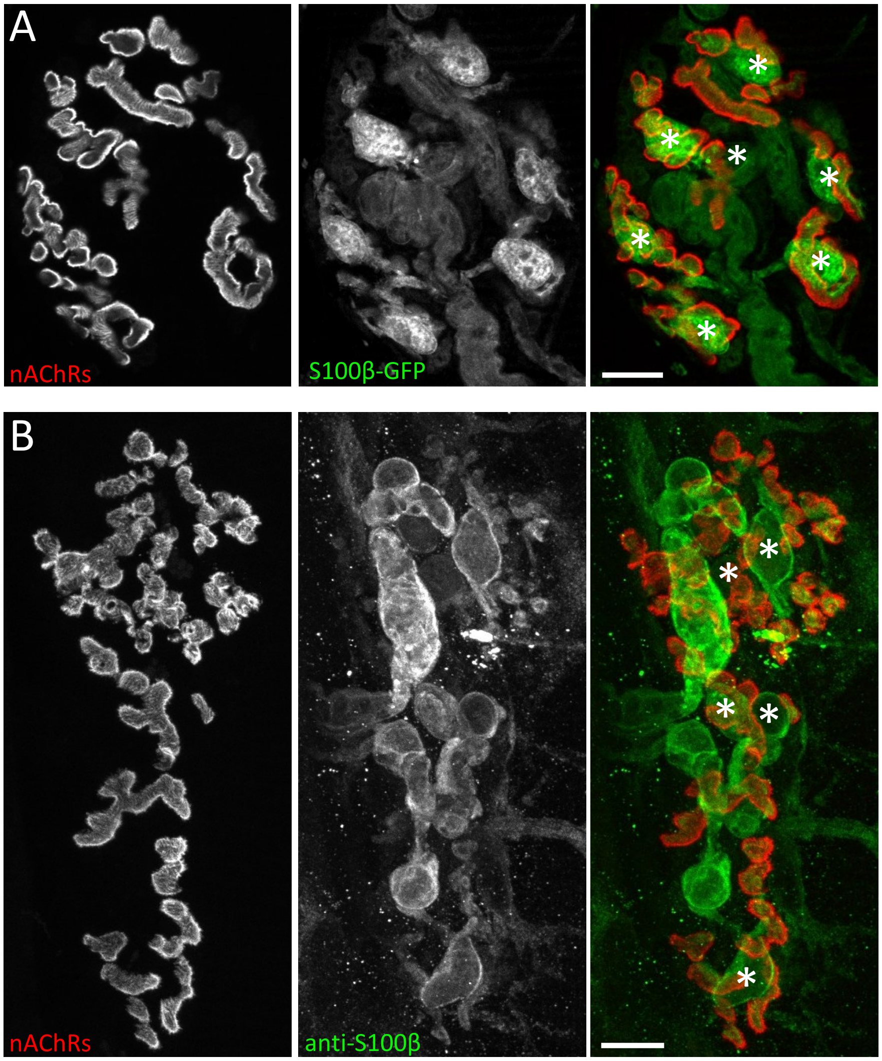

Supplementary Figure 14. PSCs remain at sternomastoid NMJs into old age. (A) Sternomastoid NMJ from old male S100β-GFP mouse. (B) Sternomastoid NMJ from old male WT mouse stained with anti-S100β antibody. PSCs (asterisks) can clearly be seen covering the acetylcholine receptors. 10 µm scale bars.

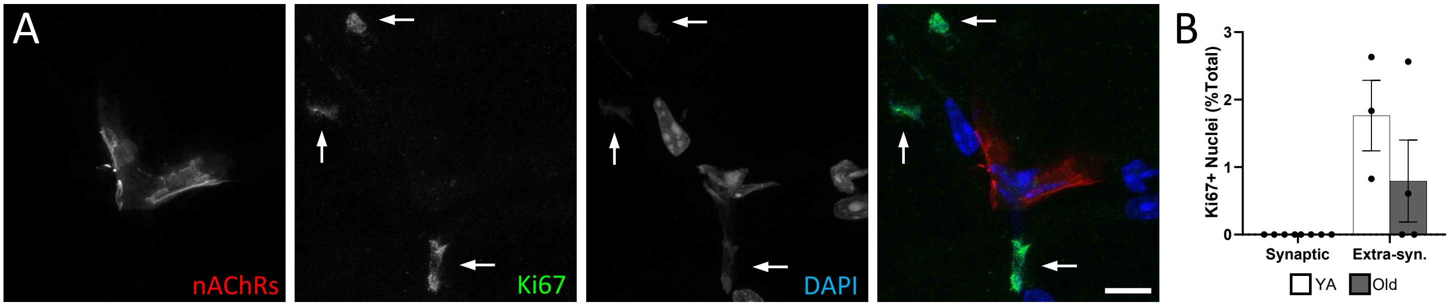

Supplementary Figure 15. Ki67-positive PSCs are absent at the NMJ. (A) Representative images of Ki67 (green) IHC in young adult TA cross section in which nAChRs are labeled with fBTX (red) and nuclei are labeled with DAPI (blue). (B) Percentage of nuclei labeled with Ki67 (arrows) in young adult or old TA muscle. Nuclei were categorized as “Synaptic”” if they lied adjacent to the fBTX stain and all other nuclei were categorized as “Extra-synaptic”. Unpaired 2-sided t-test. Values represented as mean ± SEM; n = 3-4. Scale bar = 10 μm.
