# Supplementary material for: Cellular and molecular evidence that synaptic Schwann cells contribute to aging of mouse neuromuscular junctions
Source: Aging Cell. 2023 Sep 28;22(11):e13981. doi: 10.1111/acel.13981 (PMC10652323; doi:10.1111/acel.13981)
Supplement: Supplementary file 2 — Tables S1–S2 [file ACEL-22-e13981-s001.pdf]

Supplementary Table 1. Old SC DEGs identified in other studies.

Gene	Read Count	log2 FC vs young SC	p-value	Reference
Cybb	15483.41	1.102597	0.041861139	(Verdier et al., 2012)
Tnxb	10621.51	-1.81268	0.027581426	(Verdier et al., 2012)
Mpeg1	6772.199	2.083542	0.001261864	(Verdier et al., 2012)
Clec7a	4275.218	1.429666	0.034235293	(Verdier et al., 2012)
Ccl8	4053.56	2.789377	<0.0001	(Verdier et al., 2012)
Cfb	3921.807	1.67177	0.000557927	(Verdier et al., 2012)
Efemp2	2032.541	-1.42965	0.008981677	(Verdier et al., 2012)
Agpat4	1687.745	-1.21694	0.004404667	(Verdier et al., 2012)
Cd274	1203.697	1.654163	0.011415664	(Verdier et al., 2012)
Ifi47	1200.834	1.569063	0.000325352	(Verdier et al., 2012)
Tlr13	1180.075	1.652889	0.002367042	(Verdier et al., 2012)
S100a8	286.6353	6.354282	0.019733296	(Verdier et al., 2012)
S100a9	190.2979	6.537318	0.001103747	(Verdier et al., 2012)
Col3a1	75630.87	-2.18346	<0.0001	(Painter et al., 2014)
Col1a2	27984.84	-2.02834	<0.0001	(Painter et al., 2014)
Col1a1	22430.61	-2.07517	<0.0001	(Painter et al., 2014)
Mfap5	21761.55	-2.03275	0.009187091	(Painter et al., 2014)
Col6a1	11932.78	-1.38679	0.028750326	(Painter et al., 2014)
Col6a2	6172.605	-1.27614	0.044538465	(Painter et al., 2014)
Spon2	1067.843	-1.65701	0.009887987	(Painter et al., 2014)
Itm2a	1038.862	-1.70748	0.000418984	(Painter et al., 2014)

Supplementary Table 2. Repair SC genes.

Gene	SC Log_2 FC (Old v YA)	$p < 0.05$?	PSC Log_2 FC (Old v YA)	$p < 0.05$?	Reference
Artn	0.68	N/A	-1.68	No	(K. R. Jessen & Mirsky, 2016)
Bdnf	0.35	No	-0.03	No	(K. R. Jessen & Mirsky, 2016)
Ccl2	-0.04	No	1.53	No	(K. R. Jessen & Mirsky, 2016; Ma et al., 2018)
Ccnd1	0.30	No	-0.57	No	(Atanasoski et al., 2001; Kim et al., 2001)
Epop	-0.76	No	1.35	No	(K. R. Jessen & Mirsky, 2016)
Epor	0.02	No	2.02	No	(K. R. Jessen & Mirsky, 2016)
Fgf5	1.22	No	1.64	No	(Barrette et al., 2010)
Gdnf	-0.75	No	0.77	No	(K. R. Jessen & Mirsky, 2016)
Gfap	0.17	No	1.81	No	(K. R. Jessen & Mirsky, 2016)
Il1a	0.95	No	3.39	N/A	(K. R. Jessen & Mirsky, 2016)
Il1b	0.28	No	3.37	N/A	(K. R. Jessen & Mirsky, 2016)
L1cam	0.52	No	-0.31	No	(K. R. Jessen & Mirsky, 2016)
Lif	0.52	No	2.08	No	(K. R. Jessen & Mirsky, 2016)
Ncam1	-0.03	No	-0.57	No	(K. R. Jessen & Mirsky, 2016)
Ngf	-1.40	Yes	0.07	No	(K. R. Jessen & Mirsky, 2016)
Ngfr	-0.12	No	0.76	No	(K. R. Jessen & Mirsky, 2016)
Ntf3	-0.83	No	-0.26	No	(K. R. Jessen & Mirsky, 2016)
Pou3f1	-1.30	No	-0.92	No	(K. R. Jessen & Mirsky, 2016)
Ptn	-1.72	Yes	-0.82	No	(K. R. Jessen & Mirsky, 2016)
Tnf	0.28	No	2.02	No	(K. R. Jessen & Mirsky, 2016)
Vegfa	-0.84	No	1.17	No	(K. R. Jessen & Mirsky, 2016)
