# Supplementary material for: Cellular and molecular evidence that synaptic Schwann cells contribute to aging of mouse neuromuscular junctions
Source: Aging Cell. 2023 Sep 28;22(11):e13981. doi: 10.1111/acel.13981 (PMC10652323; doi:10.1111/acel.13981)
Supplement: Supplementary file 3 — Appendix S1 [file ACEL-22-e13981-s002.pdf]

Supplemental Materials and Methods

Animals

Two sets of transgenic mice were bred: mice possessing both a thy1-CFP transgene (Feng et al., 2000) to label neurons and an S100β-GFP transgene (Zuo et al., 2004) to label all Schwann cells (S100β-GFP;thy1-CFP) on a C57BL/6 background, or mice possessing both the S100β-GFP transgene as well as an NG2-DsRed transgene (Zhu et al., 2008) to label PSCs at the exclusion of other SCs (S100β-GFP;NG2-DsRed) on a mixed background. The S100β-GFP;thy1-CFP mice were a generous gift from the Wes Thompson lab. We also used male C57BL/6 young adult mice for immunohistochemistry. "Young adult" mice used for these experiments were 3-5 months old, "middle aged" mice were 17 months old, and "old" mice were 23-29 months old. Animals were housed in a 12 h light/dark cycle and provided water and food ad libitum. Housing, breeding and experiments were performed in specific pathogen free environment. All experiments were carried out under NIH guidelines and Brown University (Protocol# 19-05-0013) Institutional Animal Care and Use Committee guidelines.

Immunohistochemistry

For analysis of PSC morphology, mice were perfused intracardially with 4% paraformaldehyde (PFA) and post-fixed in 4% PFA overnight prior to dissection of muscles. Soleus, extensor digitorum longus (EDL), and sternomastoid (STM) muscles were incubated at 4°C overnight in Alexa Fluor 555 conjugated α-bungarotoxin (fBTX, 1mg/mL, Invitrogen #B35451) and DAPI (4',6-Diamidino-2-Phenylindole, Dihydrochloride, Thermo Fisher D1306, 300 μM), each diluted 1:500 in 0.5% Triton X-100 in PBS. Muscles were washed 3X in PBS before being mounted with Vectashield (Vector Labs H-1000). Soleus muscles, STM muscles, and the largest compartment of the EDL were "filleted": the outermost layers of myofibers were cut away from the muscle and mounted on the slide. All other compartments of the EDL were whole-mounted. For S100β and NG2 staining, the muscle tissue was incubated in blocking buffer (5% goat serum, 3% bovine serum albumin, 0.5% Triton X-100 in PBS) at 4°C for 30 minutes. Rabbit anti-S100β primary antibody (DAKO Z0311, diluted 1:400) and guinea pig anti-NG2 primary antibody (courtesy of Dwight Bergles lab, diluted 1:250) was added in blocking buffer and incubated overnight at 4°C. The tissue was then washed 3 times for 5 minutes with blocking buffer and incubated with an appropriate combination of DAPI, Alexa Fluor-568 goat anti-rabbit (ThermoFisher A-21069), Alexa-Fluor-488 goat anti-rabbit (ThermoFisher A-11008), Alexa Fluor-555 goat anti-guinea pig (ThermoFisher A-21435), Alexa Fluor-488 α-bungarotoxin (Invitrogen # B13422), or Alexa Fluor-647 α-bungarotoxin (Invitrogen # B35450) in blocking buffer for 2 hours at 4°C. The tissue was then washed 3 times for 5 minutes with PBS and mounted with Vectashield.

For Ki67, CCL4, CCL7, F4/80, and CXCL16 immunohistochemistry, tibialis anterior (TA) muscles were dissected from euthanized young adult and old mice, sliced in half with a razor blade, and immediately fresh frozen in OCT on dry ice. Using a Leica CM1860 cryostat, 16 μm thick TA cross sections were taken and mounted onto gelatin coated glass slides. Four young adult cross sections were mounted on the left side and four old cross sections were mounted on the right side of each slide. The perimeter of the slides was outlined with Super PAP Pen Liquid Blocker (IHC World) and the cross sections were washed with cold 1X PBS on ice for 5 minutes. The cross sections were then fixed with cold 4% PFA on ice for 10 minutes, incubated in ice cold 100% methanol for 1 minute (except for Ki67 and CXCL16 staining), and washed with cold 1X PBS on ice 3 times for 5 minutes. The cross sections were incubated in blocking buffer (5% donkey serum

or 5% goat serum, 3% BSA, 0.1% Triton X-100 in PBS) at 4°C for 2 hours. Primary antibody solution was added in blocking buffer and incubated overnight at 4°C. Primary antibodies included Ki67 (Anti-Ki67 antibody, Abcam ab15580, diluted 1:400), CCL4 (AF-451-SP, R&D Systems, 1:100 dilution), CCL7 (LS-C295411-100, LS Bio, 1:100 dilution), F4/80 (MCA497RT, Bio-Rad, 1:250 dilution), CXCL16 (MAB503-SP, R&D Systems, 1:100 dilution), and S100β (S2532, Millipore Sigma, diluted 1:1000). The slides were then washed 3 times for 5 minutes with blocking buffer and incubated with 1:1000 DAPI, 1:1000 secondary antibody, and fBTX in blocking buffer for 2 hours at 4°C. Secondary antibodies included Alexa Fluor-488 anti-mouse IgG1 (Life Technologies A21121), Alexa Fluor-647 conjugated anti-mouse IgG1 (Life Technologies A21240), Alexa Fluor-594 conjugated anti-goat (Molecular Probes A-11058), Alexa Fluor-568 anti-rabbit (Life Technologies A11011), Alexa Fluor-555 anti-rat (Life Technologies A21434), and Alexa Fluor-647 conjugated anti-rabbit (Jackson Immunoresearch 711-605-152). The slides were washed 3 times for 5 minutes with PBS and mounted with Vectashield.

<u>Light Microscopy</u>

Images of S100β-GFP;thy1-CFP for morphological analysis were taken using a Zeiss LSM 880 confocal microscope with 405, 440, 488, and 561 nm laser lines and Zeiss Zen software. Confocal stacks were taken using a 40x, 1.3 numerical aperture oil immersion objective. NMJs were selected using the fBTX stain to find *en face* NMJs. Z-stacks and imaging parameters were then optimized to capture the entirety of each selected NMJ, including all associated DAPI, CFP, and GFP signal. Maximum intensity projections from the resulting z-stack were generated and analyzed using ImageJ.

Images of immunostained whole mounts and TA cross sections were obtained with a Zeiss LSM 900 confocal microscope with 405, 488, 561, and 640 nm laser lines using a 63x, 1.4 numerical aperture oil immersion objective and Zeiss Zen software. For the cross sections, Z-stacks were set with 1 μm step intervals. NMJs were selected using the fBTX stain to find NMJs oriented perpendicular to the cross section. The NMJs were positioned to be centered in the image. If more than one NMJ was in the field of view, the image was positioned so that both NMJs could be fully visualized. Z-stack images used for Ki67 analysis were 101.4 μm x 101.4 μm x 13 μm. Z-stack images used for CCL4, CCL7, F4/80, and CXCL16 analysis were 202.8 μm x 202.8 μm x 28 μm.

<u>Transmission Electron Microscopy</u>

One young adult (3-month-old) male mouse and one old (29-month-old) male mouse were perfused transcardially with 0.1 M sodium cacodylate buffer, pH 7.4 at room temperature (RT), followed by the same buffer with 2% PFA and 3% glutaraldehyde. EDL muscles were dissected in buffer and the largest EDL compartment was fixed overnight at RT in 2% PFA/3% glutaraldehyde in buffer. The EDL compartments were then cut in half perpendicular to their length with a razor blade near the endplate band. These muscle pieces were washed in buffer and then stained in 1% osmium tetroxide (Sigma Aldrich, 75632), 1% ferrocyanide (Sigma Aldrich, P3289) in buffer for 5 hours at RT. The muscles were washed and then stained with 1% uranyl acetate for 2 hours at RT. Muscles were dehydrated in graded ethanol at 30%, 50%, 70%, 90%, 95%, and 100% for 20 minutes at each step. After three 10-minute washes in 100% ethanol, the muscles were embedded in SPURR Low Viscosity Embedding Kit, Hard Mix (EMS, Cat #14300). A Leica EM UC7 Ultramicrotome was used to trim the blocks with a razor blade and a diamond knife, and then a diamond knife was used to obtain more than 40 individual 100 nm cross sections

per sample, which were mounted on bare 200 mesh or 300 mesh copper grids. The grids were imaged under a Philips EM410 Transmission Electron Microscope with a NANOSPRT5 camera and AMT V701 software. Digital images of individual NMJs were captured from sections at magnifications between 10,400x and 31,000x. In total, 17 images were taken of young adult NMJs, and 13 images were taken of old NMJs. It was not possible to determine which of these images were of different NMJs or whether images were of the same NMJ at different depths. The muscle, axon terminal, and PSCs in each image were identified by morphology and reviewed by a second analyst. Adobe Photoshop 2022 (version 23.2) was used to highlight and shade in each of these cell types red, blue, and green, respectively.

Image Analysis

For morphological analyses, 23-41 NMJs (technical replicates) per muscle (biological replicate) were analyzed. NMJs were selected by the presence of $fBTX^{+}$ stained nAChR clusters in an *en face* orientation. After selection of an NMJ, the entirety of the CFP and GFP signal associated with that NMJ were imaged. A cell (soma location confirmed with DAPI stain) was determined to be a PSC based primarily on its expression of the S100β-GFP transgene and by maintaining a close association with the post-synaptic nAChRs. Occasionally, the cell body of an S100β-GFP positive cell would reside just apart from the fBTX-stained nAChRs but had processes continuous with the rest of the PSC processes of the NMJ and was in a similar focal plane to other PSCs and the receptors. These cells, too, were counted as PSCs. If a GFP positive cell body resided away from the nAChRs and had a process that clearly led away from the NMJ, this cell was determined to be a migrating Schwann cell. A PSC process was defined as a fluorescent process extending more than 3 µm beyond the boundaries of the BTX stain. Blebs in the axon were defined as a large varicosity along the axon or on the axon terminal which exceeded its apposed nAChR cluster. An NMJ was considered to be poly-innervated if more than one CFP+ axon was continuous with the NMJ and that those CFP+ structures were determined not to be an axon terminal sprout. The percentage of innervated receptor area was determined by measuring the total area of fBTX signal for each NMJ, and subtracting the area of fBTX signal that did not align with a CFP+ axon terminal (i.e. subtracting the area of denervated receptors). An escaped fiber bridge was identified as a GFP+ process (usually associated with a CFP+ process) that connected two distinct NMJs. Nearest neighbor regularity index (NNRI) was based on the distance between each PSC and its closest neighboring PSC at its NMJ. The average nearest distance for every PSC analyzed within a given muscle was divided by its standard deviation to calculate the NNRI for PSCs within that muscle (Cook 1996). Discrete, non-touching fBTX stained nAChR segments were defined as an island for nAChR fragmentation analysis, and an EDL or soleus NMJ was defined as “fragmented” if it had 5 or more distinct islands. Junctional area was measured by drawing a perimeter around all receptor islands of an NMJ and calculating the resulting area. All counting and measurements of length and area were performed with ImageJ.

For Ki67 analysis, nuclei were identified by DAPI. NMJs were identified by fBTX stained nAChRs. Nuclei were categorized as “Synaptic”” if they lied adjacent to the fBTX stain and all other nuclei were categorized as “Extra-synaptic”. Nuclei were characterized as Ki67 positive if they showed signal throughout the nucleus.

CCL4, CCL7, F4/80, and CXCL16 intensity analysis was performed on TA cross sections co-stained with fBTX and anti-S100β. S100β-positive PSCs were identified by their proximity to fBTX-labeled nAChRs. S100β-positive PSCs were outlined and the signal intensity of both S100β and the protein of interest was measured using the RGB-measure plugin in ImageJ. The signal

intensity of the protein of interest was divided by S100β signal intensity to normalize for inter-sample variability in IHC efficiency. Signal intensity values are presented as a proportion of the average young adult signal intensity for each protein of interest. An average of 13.5 NMJs per animal was analyzed.

Fluorescence activated cell sorting (FACS)

Both TA and EDL muscles were collected from each of six female 4-5 and 23–24 month old S100β-GFP;NG2-DsRed mice. Muscles were dissociated in 2 mg/mL collagenase II (Worthington Chemicals, Lakewood, NJ) followed by mechanical trituration. After creating a single cell suspension with a 40 µm filter, excess debris was removed from the suspension by centrifugation in 4% BSA followed by a second centrifugation in 40% Optiprep solution (Sigma-Aldrich, St. Louis, MO) from which the interphase was collected. FACS was performed with a BD FACS Melody Cell Sorter (BD Biosciences). A single mouse was used for each replicate and an average of 5,000 PSCs and 31,000 SCs per replicate were collected for each cell group.

RNA-seq

RNA isolation and RNA-seq on FACS-isolated cells were performed by Genewiz on 6 replicates per cell type at a sequencing depth of 30 million reads per sample. RNA-seq data were trimmed for both adaptor and quality using Trimmomatic v.0.36 (Bolger et al., 2014) and the Nextera paired-end adapter. Salmon v.0.11.3 was used to index, align, and quantify transcripts (Patro et al., 2017). QC summary statistics were examined using FastQC v0.11.5 and MultiQC v1.0 (Ewels et al., 2016). Following successful alignment, R statistical software v4.1.2 was used to generate lists of differentially expressed genes.

Biomart was used to convert ensemble transcript IDs to gene IDs (Durinck et al., 2009). Tximport was used to import Salmon quantification files (Soneson et al., 2016). DESeq2 was used to determine differentially expressed genes (M. I. Love et al., 2014). Count reads equal to or less than 5 were filtered from the table before running DESeq2. EnhancedVolcano was used to generate volcano plots. Functional and pathway analysis was performed using Ingenuity Pathway Analysis (QIAGEN Inc, https://www.qiagenbio-informatics.com/products/ingenuity-pathway-analysis). Part of this research was conducted using computational resources and services at the Center for Computation and Visualization, Brown University.

Supplemental References

Bolger, A. M., Lohse, M., & Usadel, B. (2014). Trimmomatic: A flexible trimmer for Illumina sequence data. Bioinformatics, 30(15), 2114–2120. https://doi.org/10.1093/bioinformatics/btu170

Durinck, S., Spellman, P. T., Birney, E., & Huber, W. (2009). Mapping identifiers for the integration of genomic datasets with the R/ Bioconductor package biomaRt. Nature Protocols, 4(8), 1184–1191. https://doi.org/10.1038/nprot.2009.97

Ewels, P., Magnusson, M., Lundin, S., & Käller, M. (2016). MultiQC: Summarize analysis results for multiple tools and samples in a single report. Bioinformatics, 32(19), 3047–3048. https://doi.org/10.1093/bioinformatics/btw354

Love, M. I., Huber, W., & Anders, S. (2014). Moderated estimation of fold change and dispersion for RNA-seq data with DESeq2. Genome Biology, 15(12), 550. https://doi.org/10.1186/s13059-014-0550-8

Patro, R., Duggal, G., Love, M. I., Irizarry, R. A., & Kingsford, C. (2017). Salmon provides fast and bias-aware quantification of transcript expression. Nature Methods, 14(4), 417–419. https://doi.org/10.1038/nmeth.4197

Soneson, C., Love, M. I., & Robinson, M. D. (2016). Differential analyses for RNA-seq: Transcript-level estimates improve gene-level inferences. F1000Research, 4. https://doi.org/10.12688/F1000RESEARCH.7563.2
